# Piezoelectric Energy Harvester Technologies: Synthesis, Mechanisms, and Multifunctional Applications

**DOI:** 10.1021/acsami.3c17037

**Published:** 2024-05-13

**Authors:** Qinrong He, Joe Briscoe

**Affiliations:** † School of Engineering and Material Science, Queen Mary University of London, London E1 4NS, the United Kindom

**Keywords:** piezoelectricity, energy harvesters, device architectures, nanostructures, piezoelectric materials synthesis, flexible electronics

## Abstract

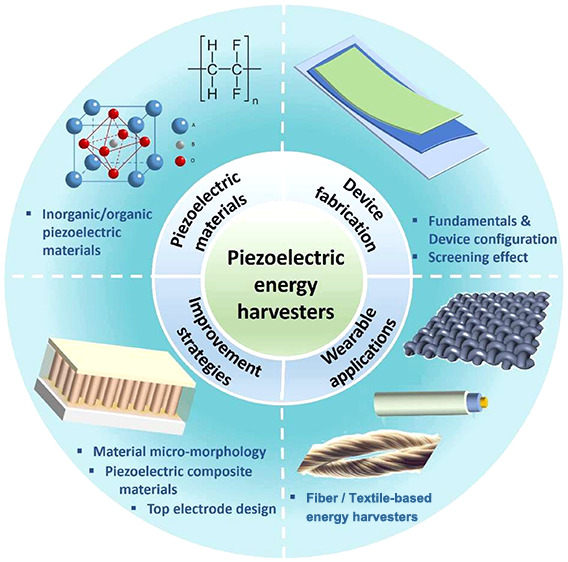

Piezoelectric energy harvesters have gained significant attention in recent years due to their ability to convert ambient mechanical vibrations into electrical energy, which opens up new possibilities for environmental monitoring, asset tracking, portable technologies and powering remote “Internet of Things (IoT)” nodes and sensors. This review explores various aspects of piezoelectric energy harvesters, discussing the structural designs and fabrication techniques including inorganic-based energy harvesters (i.e., piezoelectric ceramics and ZnO nanostructures) and organic-based energy harvesters (i.e., polyvinylidene difluoride (PVDF) and its copolymers). The factors affecting the performance and several strategies to improve the efficiency of devices have been also explored. In addition, this review also demonstrated the progress in flexible energy harvesters with integration of flexibility and stretchability for next-generation wearable technologies used for body motion and health monitoring devices. The applications of the above devices to harvest various forms of mechanical energy are explored, as well as the discussion on perspectives and challenges in this field.

## 1 Introduction

The Internet of Things (IoT), robotics, artificial intelligence (AI), and big data have drawn significant research attention, which will bring us a huge revolution into many aspects of our daily life. As a network of connected computing devices, the IoT is expected to have the ability of real-time location tracking, monitoring our body movement or health condition, such as wearable displays and wireless health tracking devices. In this regard, piezoelectric energy harvesters have emerged as a promising technology with good potential to convert ambient mechanical movement to electricity by exploiting the piezoelectric effect, presenting an attractive solution for sensors, powering low-power IoT devices, and reducing the reliance on conventional power sources.

Over the past few years, a large number of piezoelectric materials have been reported for energy harvesting applications in self-powered sensors and wearable electronics, such as zinc oxide (ZnO), barium titanate (BaTiO_3_), and lead zirconate titanate (PZT). Despite that, with increasing development of portable/wearable electronic devices such as smart watches, health, and activity monitors, it is particularly desirable to research a flexible energy harvester that can capture multiple forms of mechanical energy with enhanced energy conversion efficiency, which holds great promise in personal smart devices. To meet the requirement of flexibility and comfort, a number of flexible substrates with their unique properties of lightweight, comfort, softness and wearable convenience hold great potential to be used as platform to be integrated with piezoelectric materials used as portable/wearable electronic devices, which can generate energy from jumping, joint bending, and running etc.

In this regard, a multitude of scientific papers have been reported investigating the various range of energy harvesters using different strategies to obtain higher output performance with high flexibility. In this review, the basic working principle and classifications are discussed. We also cover the recent research into different piezoelectric materials, material and device fabrication and measurement methods. Strategies for improving the energy harvesting performance are also investigated. The current challenges and future directions in their development are summarized, which can be used as reference and an introduction to the energy harvester field to help the development of portable/wearable energy harvesters.

## 2 Principle of Piezoelectricity

**Figure 1:**
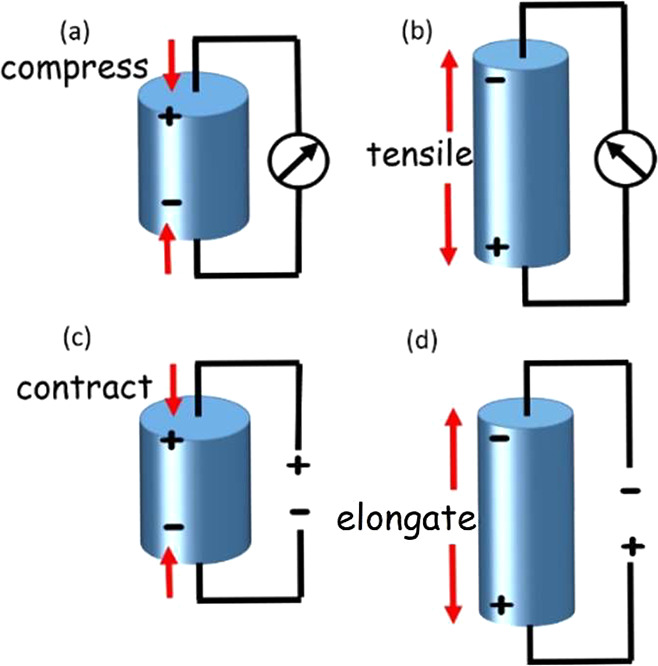
Scheme of the direct piezoelectric effect (a) and (b), where compressive and tensile forces applied to a material produce an electric field; and converse piezoelectric effect (c) and (d) where an electrical field applied to a material causes contraction and elongation, respectively.

**Figure 2:**
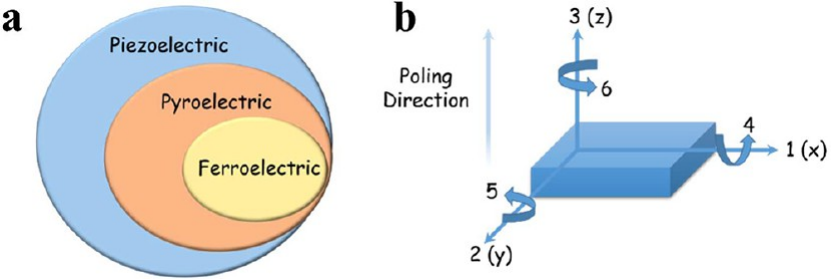
(a) The relationships of ferroelectric, pyroelectric and piezoelectric materials. (b) Schematic of a piezoelectric transducer and relevant tensor directions for defining the constitutive relations.

Piezoelectric materials are defined by their ability to generate deformation when subjected to electric field, or electric charges when subject to mechanical stress. When piezoelectric materials experience mechanical strain, they produce an electric charge known as the direct piezoelectric effect (Figure 1a,b). The converse piezoelectric effect occurs when an applied electric field causes mechanical stress in piezoelectric materials. (Figure 1c,d). In general, piezoelectric materials lack a centrosymmetric structure. When an external mechanical force is applied on a piezoelectric material, the positive and negative centers of the material will be separated, resulting in an electric dipole moment. Among the 32 crystallographic point groups, it is expected that 21 noncentrosymmetric point groups are able to exhibit piezoelectric properties.^1^ Many research studies have been conducted to take advantage of piezoelectric materials on a variety of practical applications. The converse piezoelectric effect is mostly used in the field of acoustic emitters, vibration damping and actuators.^1,2^ Various energy harvesting research has been conducted to use mechanical energy to create useful electricity by using the direct piezoelectric effect. In addition, some piezoelectric materials also exhibit some other unique properties, such as pyroelectricity and ferroelectricity. The relationship between ferroelectricity, pyroelectricity, and piezoelectricity is shown in Figure 2a. As shown, all ferroelectric materials are also pyroelectric and piezoelectric, and all pyroelectric materials are also piezoelectric, but not all piezoelectric materials are either pyroelectric or ferroelectric. When a piezoelectric material is subjected to compression, a dipole and net polarization are produced in the direction of the applied stress. In addition, there is spontaneous polarization within the pyroelectric and ferroelectric materials. For pyroelectric materials, the spontaneous polarization can be changed due to a change in temperature. For ferroelectric materials, spontaneous polarization is reversible in response to the application of external electric fields.^3^ Materials possessing multiple properties will hold great promise for energy harvesting from a variety of factors, including light, temperature changes, impact, and vibration.

The direct and converse piezoelectric effect can be expressed by the piezoelectric constitutive eqs 1 and 2, respectively as follows:^4^
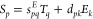
1
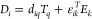
2where *S*_*p*_ and *T*_*q*_ are the strain and stress in *p* and *q* directions, respectively; *D*_*i*_ and *E*_*k*_ are the displacement and electric field in *i* and *k* directions, respectively; *s*_*pq*_^*E*^ and ε_*ik*_^*T*^ are the elastic compliance tensor and dielectric constant tensor under constant electric field and stress, respectively; and *d* is the piezoelectric constant tensor. As shown in Figure 2b, the numbers “1”, “2”, and “3” correspond to the *x*, *y*, and *z* axes. The indices “4”, “5”, and “6” refer to the shear planes, which are perpendicular to the directions “1”, “2”, and “3” respectively. The piezoelectric charge coefficient abbreviated as *d*_*xy*_ relates the electric charge generated per unit area with an applied mechanical force in the unit of Coulomb/Newton (C/N) (the ratio of open circuit charge density to the applied stress), where *x* and *y* represent the direction of the induced polarization and applied stress, respectively.^5^ A piezoelectric material has a polar axis that depends on the crystal orientation or the poling direction. Figure 2b illustrates this by designating the polar axis as the “3” direction and the opposite direction, which is at a right angle to the polar axis, as the “1” direction. When the applied stress is along the direction of the polar axis, it can be denoted as 33-mode (longitudinal piezoelectric coefficient),^6^ whereas the configuration when the applied stress is perpendicular to the polar axis is denoted as 31-mode (transverse piezoelectric coefficient).^7^ Generally, the *d*_33_ value is higher than the *d*_31_ value as shown in Table 1.^1,6^ The piezoelectric voltage coefficient abbreviated as *g*_*xy*_ is the ratio of the electric field produced to the applied mechanical stress in the unit of Vm/N, where *x* and *y* represent the direction of the induced electric field and applied stress, respectively, or, induced strain in the *y* direction per unit electric displacement applied in *x* direction.^8^ The relationship between *d*_*xy*_ and *g*_*xy*_ can be expressed through the below equation (ε_*xy*_ is dielectric constant, ε_0_ is vacuum permittivity), which can be analogous to the fundamental circuit equation *V* = *Q*/*C*, where *V* is voltage, *Q* is charge, *C* is capacitance, suggesting an increase in *d*_*xy*_ often coupled with an increase in dielectric constant when the *g*_*xy*_ coefficient remains relatively constant.^9^
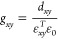
In addition, the electromechanical coupling factor, *k*_*xy*_, serves as an indicator of the efficiency with which a piezoelectric material transforms mechanical energy into electrical energy or vice versa, where *x* denotes the direction along which the electrodes are applied; *y* denotes the direction along which the mechanical energy is applied, or developed.^10^ The above factors are crucial in various applications such as sensors, actuators, and transducers where the conversion between electrical and mechanical energy is essential. Table 1 shows record values for piezoelectric properties of some piezoelectric materials.

**Table 1:** Some Piezoelectric Materials and Their Main Piezoelectric Properties

	piezoelectric coefficient (*d*)		electromechanical coupling factor (*k*)	
piezoelectric materials	*d*_31_	*d*_33_	*k*_31_	*k*_33_
BaTiO_3_ single crystal^11^	–34.5 pC/N	85.6 pC/N	0.315	0.56
BaTiO_3_ ceramic^12^	–79 pC/N	191 pC/N	0.49	0.47
LiNbO_3_ single crystal^12,13^	–1 pC/N	6 pC/N	0.02	
LiTaO_3_ single crystal^14^	–3 pC/N	9.2 pC/N		
PZT-5A ceramic^15^	–171 pC/N	374 pC/N	0.34	0.7
PZT-5H ceramic^16^	–274 pC/N	593 pC/N		0.75
PVDF^17^	17.9 pC/N	–27.1 pC/N	10.3	12.6
PVDF-HFP^17^	30–43 pC/N	24 pC/N	0.187	0.36
Bulk ZnO^18,19^	5 pC/N	12.4 pC/N	0.18	0.47
ZnO nanorods (NRs)^20^		49.7 pm/V		

## 3 Piezoelectric Materials

Some of the earliest discovered piezoelectric materials are quartz and Rochelle salt, which were used in ultrasonic applications during the 1900s.^10^ Various kinds of piezoelectric materials have been found or synthesized with good piezoelectric coefficient and chemical stability during the years of development, which have been applied in different fields.^21−24^ Piezoelectric materials can be separated into the two categories of inorganic and organic piezoelectric materials.

### 3.1 Inorganic Piezoelectric Materials

#### 3.1.1 Wurtzite Structure

**Figure 3:**
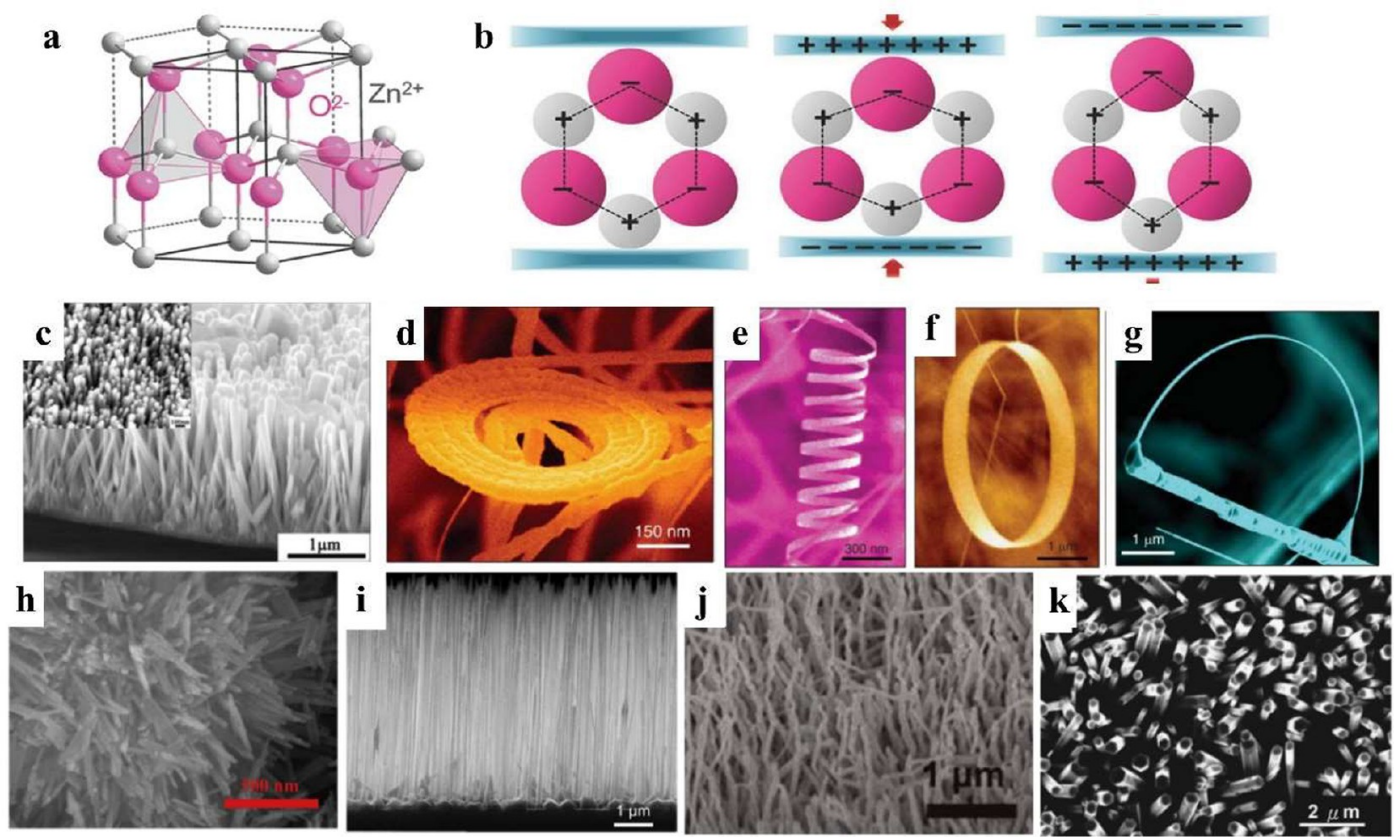
Piezoelectrical materials with wurtzite structure and their different morphologies. (a) Structural and atomic model of wurtzite structure ZnO. (b) The displacement of the center of positive charge from that of the negative charge under the compression and tension of an external force. Reproduced with permission from ref (25). Copyright 2017 John Wiley and Sons. A collection of novel ZnO nanostructures (c) nanowires (NWs). Reproduced with permission from ref (26). Copyright 2012 American Chemical Society. (d, e) Nanospirals and nanosprings. Reproduced with permission from ref (27). Copyright 2003 American Chemical Society. (f) Nanorings. Reproduced with permission from ref (28). Copy 2004 Elsevier. (g) Nanobows. Reproduced with permission from ref (29). Copyright 2004 American Chemical Society. (h) ZnS NWs. Reproduced with permission from ref (30). Copyright 2019 Elsevier. (i) GaAs NWs. Reproduced with permission from ref (31). Copyright 2017 John Wiley and Sons. (j) GaN NWs. Reproduced with permission from ref (32). Copyright 2012 American Chemical Society. (k) CdS NWs. Reproduced with permission from ref (33). Copyright 2008 AIP Publishing.

Piezoelectric materials possessing a wurtzite structure have attracted lots of attention, such as zinc oxide (ZnO), gallium nitride (GaN), cadmium sulfide (CdS), and zinc sulfide (ZnS). This structure lacks a center of symmetry causing piezoelectricity under external mechanical forces. Since Wang’s group first reported a ZnO-based piezoelectric nanogenerator in 2006,^34^ ZnO has been widely researched in the field of energy harvesting applications. ZnO is a typical wide band gap (3.10–3.37 eV) semiconducting material,^35^ which has been demonstrated to have many applications, such as photovoltaics, piezoelectric nanogenerator and high performance sensors. As shown in Figure 3a, tetrahedrally coupled O^2–^ and Zn^2+^ ions are alternately stacked along the *c*-axis to form the hexagonal structure of wurtzite ZnO.^36^ ZnO has a noncentrosymmetric structure as a result of the tetrahedral coordination of the Zn^2+^ cations and O^2–^ anions, which is also the core of its piezoelectricity.^37^ As shown in Figure 3b, under strain-free conditions, the centers of the positive ions and negatives ions overlap with each other, where the crystal shows no polarization. The center of the cations and the center of the anions are relatively shifted if a stress is applied at the apex of the tetrahedron, which results in the formation of charges on the crystal surface of ZnO causing a piezoelectric polarization. ZnO can be easily synthesized by different methods such as hydrothermal,^38^ electrochemical deposition,^39,40^ template-assisted growth,^41,42^ sol–gel synthesis, chemical vapor deposition (CVD), and plasma enhanced chemical vapor deposition.^43−46^

By controlling the synthesis conditions, such as temperature, substrates, and synthesis methods, to adjust the electrostatic interaction energy and distinct chemical activities of (0001)-Zn and (0001̅)-O polar surfaces, a wide range of ZnO nanostructures can be synthesized such as NWs, nanospirals, nanosprings, nanorings, and nanobows (Figure 3c–g), and other complex nanoarchitectures. The solution-based method has become one of the most common methods for synthesizing ZnO due to its general low growth temperature (60–100 °C) with the growth solution consisting of zinc nitrate and hexamethylenetetramine (HMT), which is also compatible with the temperature limitations of polymer-based substrates such as polyethylene terephthalate film (PET) and polyethylene naphthalate (PEN) because these substrates cannot be processed about 150–200 °C.^26,47−49^ In addition to ZnO, some other wurtzite structure piezoelectric materials have the same crystal structure and analogous physical properties to ZnO, which also have attracted increasing research interests in generating electrical energy from external mechanical forces. Figure 3h-k show ZnS NWs,^30^ GaAs NWs,^31^ GaN NWs^32^ and CdS NWs, respectively. Furthermore, to improve the device performance, different strategies such as doping, different substrates and morphology of ZnO have also been investigated, which will be discussed more in sections 4.2 and 5.

#### 3.1.2 Perovskite Structure

**Figure 4:**
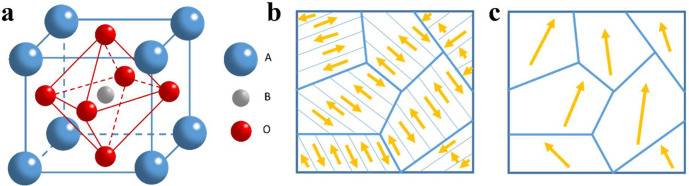
(a) Illustration of ABO_3_ perovskite structures. Schematic illustration of the grains and domain orientation of a ferroelectric material before (b) and after poling (c).

Lead zirconate titanate (PZT) and barium titanate (BaTiO_3_) are well-known for their piezoelectricity and ferroelectricity, which have been applied in sonar technology or piezo ignition for a long time.^50^ They have a perovskite structure, which has an ABO_3_ structure shown in Figure 4a, including large sized cations (A) in the corner, small sized cations (B) in the middle and the anion, commonly oxygen atoms, at the faces of the unit cell.^51,52^ Under external mechanical forces, the positive ions are displaced relative to the negative ions, which cause the breaking of the centrosymmetric structure presenting piezoelectricity and potential ferroelectricity.

PZT and BaTiO_3_ are ferroelectric materials that exhibit the piezoelectric effect due to the noncentrosymmetric crystal structures but also exhibit spontaneous polarization in the absence of electric field, which can be changed and reversed by external electric field. The primary distinction between piezoelectricity and ferroelectricity lies in the presence of spontaneous reorientable polarization. A spontaneous dipole refers to the formation of a permanent electric dipole moment in the absence of an external electric field or external stress. This phenomenon is commonly observed in certain materials with asymmetric charge distributions or noncentrosymmetric crystal structures. A spontaneous (permanent) dipole arises from an asymmetry in the positions of the positive and negative ions. The switchable polarization arises because there are multiple minima in the thermodynamic landscape of ionic positions. Therefore, the most stable state for the ions is to reside in a noncentrosymmetric position, and their position can be switched by application of sufficient electric field (above the coercive field).^53^ Ferroelectrics contain many individual regions with aligned electrical dipoles. The regions with uniform polarization directions are called domains as shown in Figure 4b. When ferroelectrics are subjected to a strong direct current (DC) electric field, as shown in Figure 4c, the small domains with random orientated electrical dipoles can be aligned in common direction to show piezoelectricity after a polarization process known as poling.

PZT, as one of the most common piezoelectric ceramics, has been widely studied for energy harvesting due to its high piezoelectric coefficient (*d*_33_ of 500–600 pC/N).^54,55^ PZT bulk ceramics or films can be synthesized by a variety of methods such as solid-state synthesis,^56,57^ molten salt,^58^ sol–gel processing,^59−61^ rf-magnetron sputtering,^62−64^ CVD,^65^ and metal organic decomposition (MOD).^66,67^ For PZT film deposition, the bottom substrate is one of the key factors to get a well-crystallized PZT film to minimize stress and defects.^68^ Therefore, there are various substrates can be used for PZT growth with high quality crystallinity, such as Pt/Ti/SiO_2_/Si,^69−71^ Al_2_O_3_/Si,^72−74^ MgO,^75−77^ fluorophlogopite mica (KMg_3_(AlSi_3_O_10_)F_2_),^78,79^ and LaNiO_3_,^80,81^ on which the homogeneous texture can provide nucleation sites facilitating the crystal orientation.

**Figure 5:**
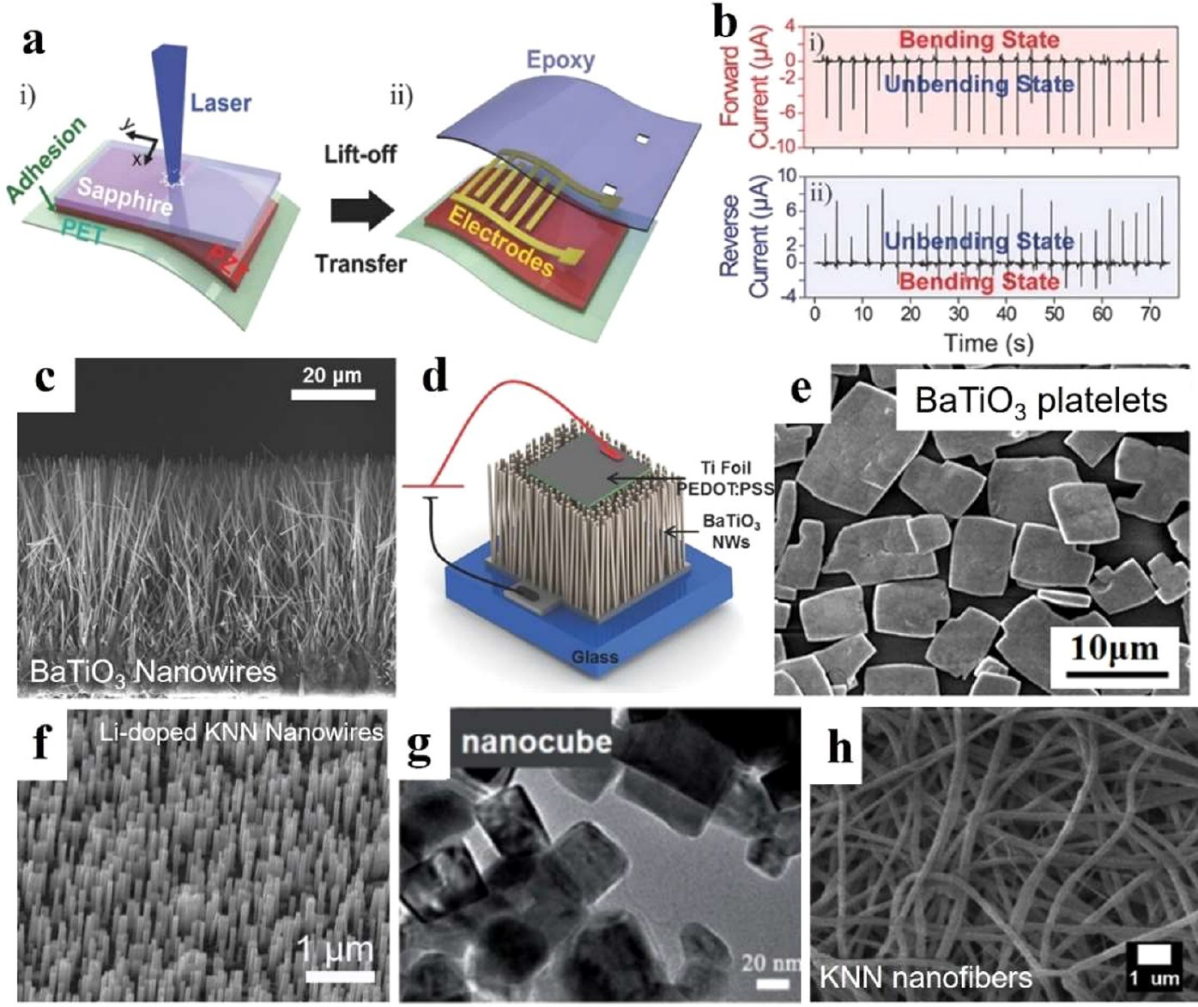
Piezoelectrical materials with perovskite structure and their different morphologies. (a) Scheme of the laser lift-off (LLO) fabricated flexible PZT thin film-based nanogenerator with (b) output current by human finger bending under forward–reverse connection. Reproduced with permission from ref (82). Copyright 2014 John Wiley and Sons. (c) SEM image of BaTiO_3_ NWs on Ti foil. (d) Scheme of PEDOT:PSS/BaTiO_3_ NWs energy harvester. Reproduced with permission from ref (83). Copyright 2014 John Wiley and Sons. (e) SEM of BaTiO_3_ platelets. Reproduced with permission from ref (84). Copyright 2015 Royal Society of Chemistry. SEM image of (f) Li-doped KNN NWs. Reproduced with permission from ref (85). Copyright 2021 Elsevier. (g) KNN nanocubes. Reproduced with permission from ref (86). Copyright 2015 Elsevier and (h) KNN nanofibers. Reproduced with permission from ref (87). Copyright 2021 Elsevier.

However, for wearable technical applications, the brittleness of PZT bulk or thin film limits the applications in flexible and stretchable operation modes. For this reason, some researchers have started to utilize flexible substrates such as indium tin oxide (ITO) coated PET, ITO/PEN, and flexible Ni–Cr metal foil substrates. A laser lift-off (LLO) procedure can be used to transfer a PZT film onto a flexible film without causing structural damage to fabricate lightweight and flexible energy harvesters. Figure 5a shows the schematic of LLO process. The working principle of laser lift-off is based on the active layer and the substrate exhibiting different absorption of the laser light. The PZT layer has a band gap of about 3.2–3.6 eV, whereas the sapphire band gap energy is about 10 eV.^82^ Short wavelength laser light passes through the sapphire, and ablates the interface when it is absorbed by the PZT, where the confined plasma at the interface results in lift-off or separation of the materials. Figure 5b shows a 3.5 cm × 3.5 cm PZT-based flexible device fabricated through LLO can generate current of ∼8.7 μA by irregular and slight bending by a human finger.^82^

However, due to the concern about element lead in PZT, some lead-free inorganic materials have garnered a lot of interest because of their nontoxic and environmentally friendly properties. BaTiO_3_, as a lead-free inorganic material, is suggested as a promising alternate material with ABO_3_-type perovskite structure.^88^ Different synthesis methods can be used to obtain BaTiO_3_, such as solid-state reaction, sol–gel processing, microwave heating, a microemulsion process, a polymeric precursor method, ball milling, and solvothermal methods.^89^ Similar to PZT, BaTiO_3_ also is ferroelectric, which needs a poling process to align the electrical dipoles to activate the electromechanical properties. BaTiO_3_ NWs have been widely investigated because 1D NWs are more sensitive to small, random mechanical disturbances.^90−92^Figure 5c shows a BaTiO_3_ NWs array synthesized on Ti foil by a two-step hydrothermal process. Figure 5d is the schematic of PEDOT:PSS/BaTiO_3_ NWs fabricated vibration energy harvester, which can generate output voltage and current of ≈775 mV and ≈1.86 nA from 0.25 g input acceleration.^83^ As shown in Figure 5e, BaTiO_3_ platelets can be spin-coated on ITO/PET generating maximum voltage and current outputs reaching 6.5 V and 140 nA by bending, respectively.^84^

Ferroelectric materials lose their spontaneous polarization because the unit cell changes shape resulting in loss of its aligned electric dipoles and thus ferroelectric behavior above the Curie temperature (*T*_c_). KNN-based materials (KNbO_3_, NaNbO_3_, Na_*x*_K_1–*x*_NbO_3_ (NKN)) have also attracted increasing attention due to their high Curie temperature (*T*_c_ = 350–475 °C),^93−95^ good electromechanical coupling factors (*k*_33_ ≈ 70%) and piezoelectric coefficients (*d*_33_ > 200 pC/N).^21,96−98^ The piezoelectric coefficient of KNN-based materials can be improved up to 490–650 pC/N, which is even comparable to those of soft lead-based ceramics used in industry.^99−101^ KNN-based materials with different nanostructures such as NWs, nanocubes and nanofibers could be obtained by hydrothermal method, normal sintering, electrospinning, etc., as shown in Figure 5f–h.^85−87^ These could be directly fabricated as an energy harvester or used as nanofillers in some organic piezoelectric materials to fabricate a composite nanogenerator.

#### 3.1.3 Two-Dimensional (2D) Materials

**Figure 6:**
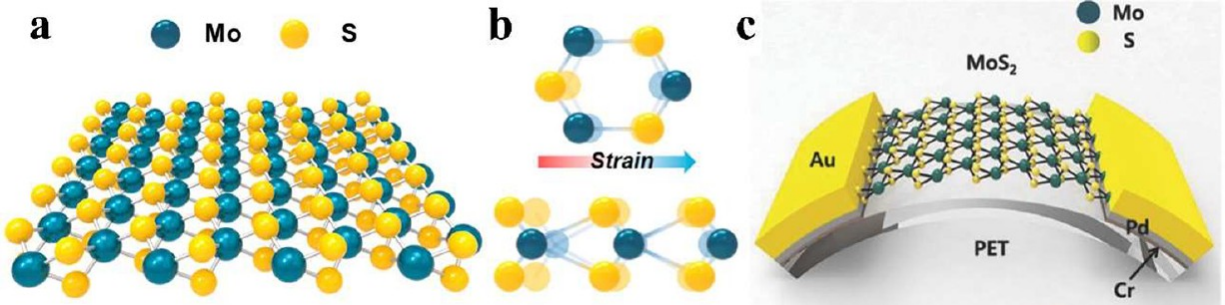
The piezoelectric structure of 2D materials MoS_**2**_. (a) Schematic of atomic structure and a single-crystalline monolayer of MoS_2_ flake. (b) Top (upper) and cross-view (lower) atomic structures for the monolayer MoS_2_ under an external stress. Reproduced with permission from ref (110). Copyright 2016 Elsevier. (c) Picture of MoS_2_-based energy harvester. Reproduced with permission from ref (112). Copyright 2014 WILEY-VCH.

Recently, monolayer MoS_2_, as a 2D nanomaterial, has been of particular interest due to its comparable in-plane stiffness to that of steel (∼270 GPa), fracture strength (∼23 GPa)^102^ and adjustable bandgap (1.2–1.8 eV),^103,104^ which has been widely used in the fields of electronic transistors, batteries and photocatalysis.^105−107^ The noncentrosymmetry and absence of inversion symmetry of the 2D crystallographic sheets certainly along the in-plane direction are the basis for piezoelectricity in MoS_2_.^108^ It is found that the oscillating piezoelectric electrical outputs only occurs when the two-dimensional crystal has an odd number of layers.^109^Figure 6a,b shows the crystal structure: Between two identical S layers, a single Mo atomic layer forms a hexagonal lattice.^110^ Two S atoms are asymmetrically occupied on the left site of each rhombic prismatic unit cell, whereas one Mo atom is occupied on the right. Therefore, an external electric field in the hexagonal lattice pointed from the S site to the Mo site can be created by stretching the Mo–S bond.^109,110^ A monolayer of MoS_2_ can be prepared by many methods such as chemical route,^111^ chemical vapor deposition (CVD) process,^112^ and liquid and micromechanical exfoliation.^113,114^Figure 6c shows the scheme of CVD-grown fabricated MoS_2_ nanosheet (NS) energy harvester on PET. The output current and voltage of MoS_2_ nanosheet device by S vacancy passivation can reach higher than pristine MoS_2_ device up to 100 pA and 22 mV, respectively.^112^

### 3.2 Organic Piezoelectric Materials

**Figure 7:**
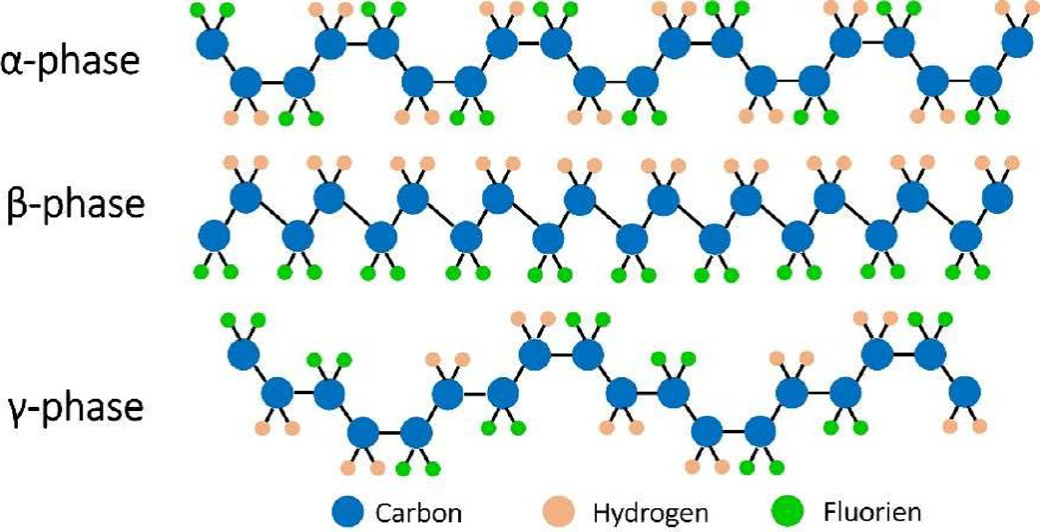
Schematic of the three α, β, and γ conformations of PVDF. Reproduced with permission from ref (115). Copyright 2018 Multidisciplinary Digital Publishing Institute (MPDI).

**Figure 8:**
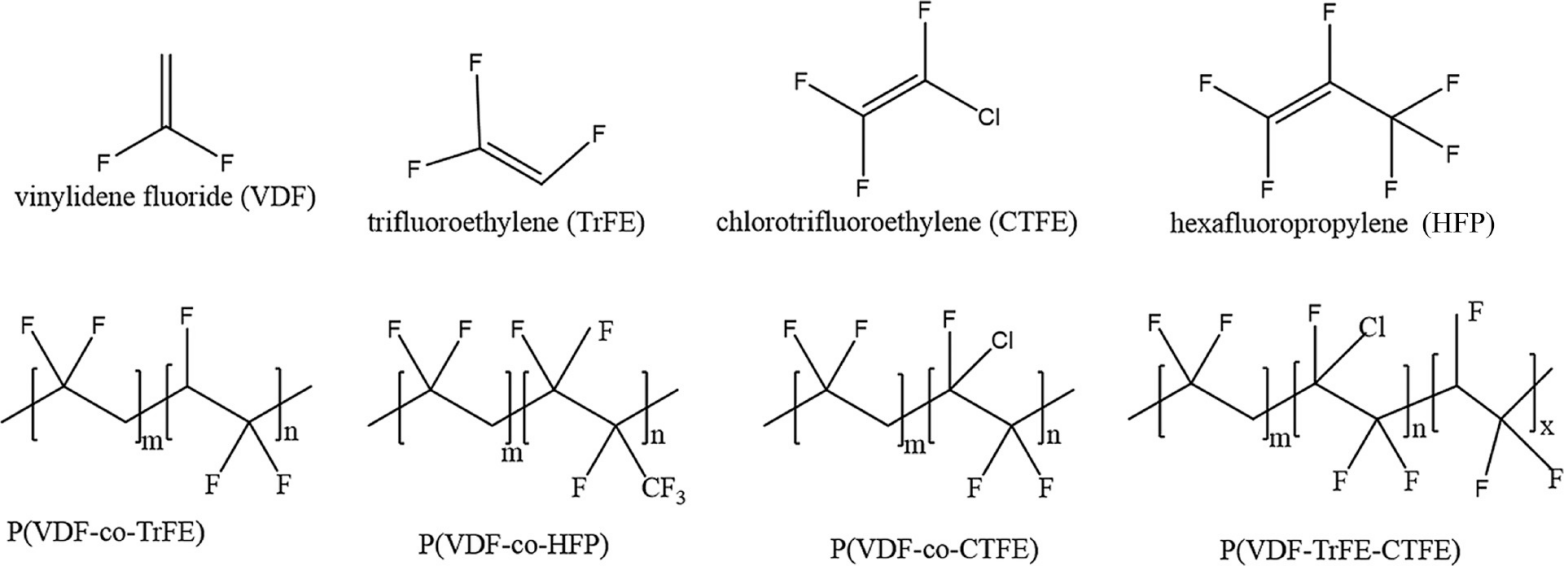
Diagrams of the molecular structure of VDF, TrFE, CTFE and HFP and the corresponding four types of copolymers. Reproduced with permission from ref (122). Copyright 2017 Royal Society of Chemistry.

Poly(vinylidene fluoride) (PVDF), as a polymeric-based piezoelectric material, has gained lots of interests for practical piezoelectric applications due to its good flexibility and biocompatibility. PVDF has the basic molecular formula (−CH_2_–CF_2_−)_*n*_, in which the fluorine atoms shows large van der Waals radius (1.35 Å, versus hydrogen 1.2 Å) and electronegativity in the polymer chain [−CH_2_–CF_2_−] leading to a dipole moment perpendicular to the chain in each monomer unit.^116^ Therefore, PVDF shows five different crystalline polymorphs based on the polymer chain structure: α, β, γ, δ, and ε.^117,118^Figure 7 shows the chain conformation for the most investigated PVDF phases: α, β and γ-phases. The polar crystalline phases of PVDF exhibit piezoelectric properties including β-phases with all-trans chain conformation (TTT) and γ-phases with intermediate conformation (T3G^+^T3G^–^).^119,120^ The α-phase is a nonpolar phase caused by the self-cancelation of dipoles resulting from the antiparallel packed trans–gauche (TG^+^TG^–^) molecules.^121^ Since the β-phase shows the strongest piezoelectricity, a variety of approaches have been developed to enhance the β-phase. One of the methods is copolymerization to adjust the polymorph structure. Figure 8 shows the molecular structure of the commonly incorporation monomers and their corresponding copolymer. The monomers including trifluoroethylene (TrFE), chlorotriuoroethylene (CTFE), and hexafluoropropylene (HFP) can facilitate the formation of ferroelectric β-phase because the addition of the second monomer unit influences the chain distance known as steric hindrance effect and reduces the activation energy for β-phase transition to facilitate the crystallization in the polymer chain.^121,122^ In addition, annealing conditions, surface charge treatment, electrical poling processes, and mechanical forces such as pressing and stretching can also be used to increase the fraction of the β-phase and the crystallinity degree to obtain higher piezoelectricity.^115,123,124^ A recent study found the press & folding (P&F) technique can form PVDF films with high β phase content (∼98%) and high breakdown strength (880 kV/mm).^125^ The tension, shearing and compression during the pressing and folding can facilitate the formation of β phase. Nanoprecipitation combined with a bisolvent phase separation technique was reported to produce PVDF nanoparticles (NPs) with a predominant piezoelectric δ-phase with piezoelectric coefficient (*d*_33_) of ca. −43 pm/V.^126^ Since PVDF has a ferroelectric structure, it is important to use high electric field to align all the dipoles with the electric field (at the nanoscale). Therefore, to generate a strong piezoelectric response (at the macroscale), electrospinning has been considered a promising technique to align the polymer dipoles with high β phase content attributed to the simultaneous high mechanical stretching and electrical poling in the process of electrospinning. During electrospinning, voltage polarity and ambient relative humidity have been shown to affect the piezoelectric performance. It has been reported that 60% humidity and negative voltage polarity can lead to a porous morphology and affect surface chemistry leading to higher performance of PVDF.^127^ By adding additives such as MgO,^128^ BaTiO_3_,^129^ ZnO,^130^ carbon nanotube (CNT),^131^ and KNN-based materials,^132^ the crystallinity and fraction of β phase can be enhanced as well because these NPs can interact with the −CH_2_ or −CF_2_ to affect the conformation of the polar phases and act as nucleating agent to help crystallinity. Moreover, the nanocomposites combining PVDF with inorganic/organic materials to fabricate energy harvesters can not only decrease the screening effect but also improve the performance, flexibility, and mechanical properties of the device, which will be discussed in the section 5.2 in details.

## 4 Nanogenerator Designs and Fabrication

### 4.1 Fundamentals and Device Configuration

The working principles of piezoelectric energy harvesters can be explained as follows: The cation and anion relative displacement in a piezoelectric material will generate piezoelectric polarization leading to a potential difference when it subjected to external force. When there is a significant enough potential difference by applying and releasing the force, it can drive charges to flow through the external circuit causing an output voltage and current to charge a battery, capacitor or use it on a load.

**Figure 9:**
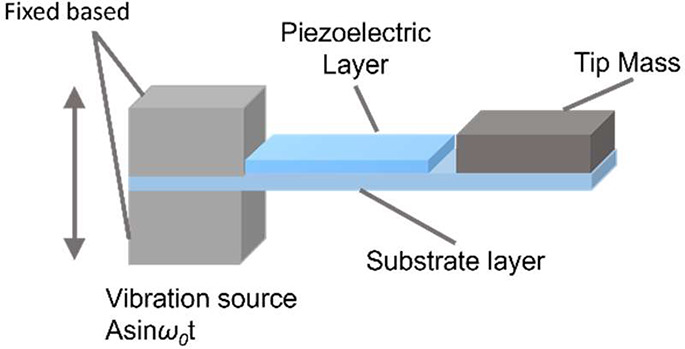
A typical cantilevered piezoelectric energy harvester device configuration.

**Figure 10:**
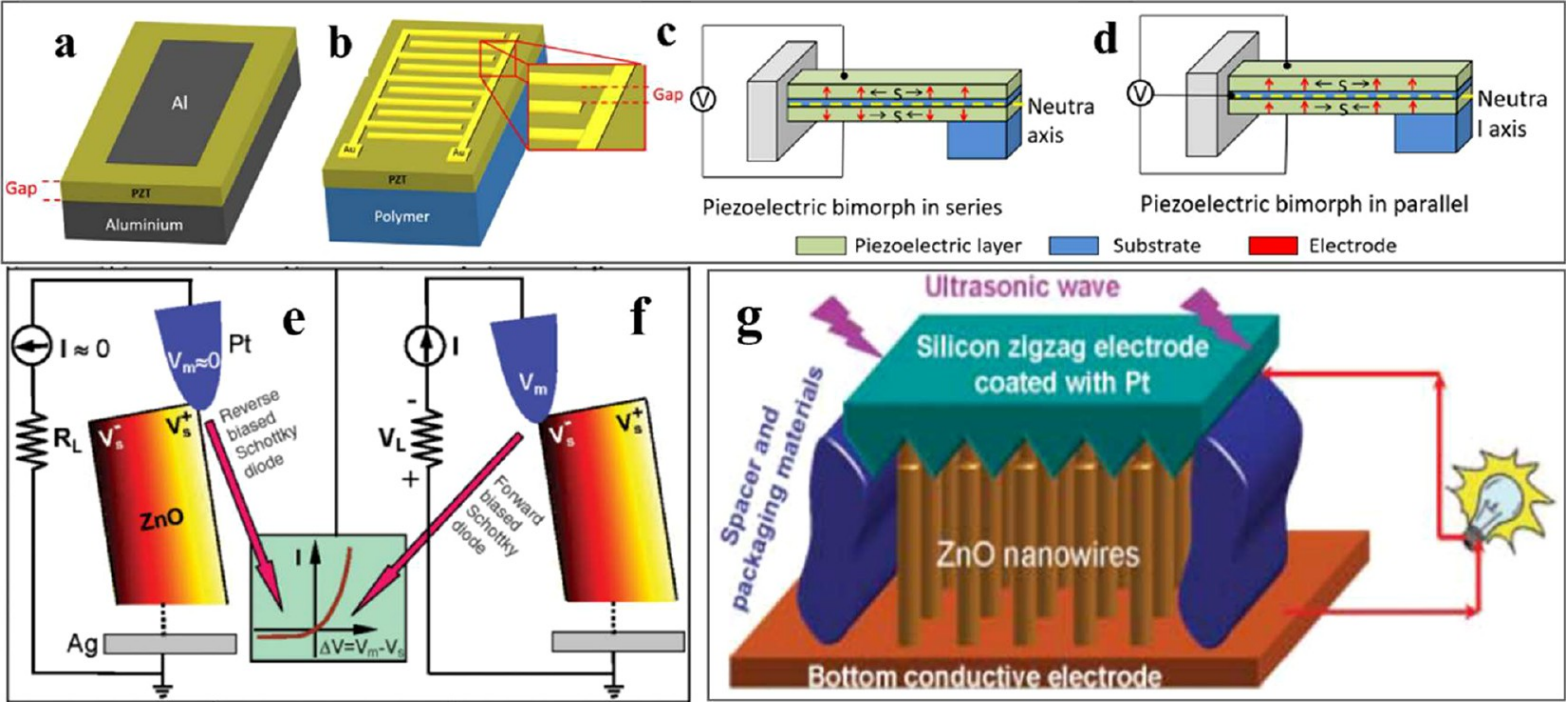
Configurations of piezoelectric energy harvesters. Diagram of unimorph cantilever configuration with different electrode shape (a) metal–insulator–metal structure (MIMs) and (b) interdigitated electrodes structure (IDEs). Reproduced with permission from ref (136). Copyright 2020 Elsevier. Schematic of bimorph cantilever in series (c) and parallel connections (d). Reproduced with permission from ref (10). Copyright 2018 AIP publishing. (e, f) Potential distribution in the NRs due to the piezoelectric effect, the contacts between the AFM tip and the semiconductor ZnO NRs in the inset box. Reproduced with permission from ref (34). Copyright 2006, The American Association for the Advancement of Science. (g) Schematic of ZnO-based nanogenerator covered by a zigzag electrode. Reproduced with permission from ref (137). Copyright 2007 The American Association for the Advancement of Science.

One of the most common device configurations is a cantilever for film or sheet piezoelectric materials as show in Figure 9, which can be used to harvest energy from vibration.^10^ Generally, a cantilever configuration comprises a continuous substrate fixed at the left end to a vibration excitation platform, and the free right end is fixed with a tip mass.^133^ A piezoelectric layer is attached to the cantilever surface under alternating deformation.^134^ The piezoelectric layer may also form the whole cantilever, without a separate substrate. The mechanical strain within the piezoelectric material in a cantilever is mostly increased at resonance, where the free end of cantilever configuration can generate the largest deformation induced by the vibration excitation resulting in the largest output performance, whereas the output performance decreases again after vibration frequency increases above resonance. Unimorph and bimorph straight cantilevers are two forms of straight cantilever as shown in Figure 10a,b and 10c,d, respectively.^10,135^ The metal–insulator–metal (MIM) structure is one of the common structures, as shown in Figure 10a. For the MIM structure, the piezoelectric layer is deposited on a conductive substrate as a bottom electrode with another conductive film coated on top as a top electrode. Instead of depositing a piezoelectric film on a conductive substrate, a piezoelectric film can be deposited on an insulating substrate only with interdigitated electrodes (IDE) on top, shown in the Figure 10b. When the energy harvester is subjected to external mechanical force, according to eq 3, the voltage generated by the buildup of charge due to polarization can be calculated as
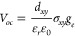
3where *d*_*xy*_, ε_*r*_, and ε_0_ are the piezoelectric coefficient, relative dielectric constant and the permittivity of vacuum, respectively, open-circuit voltage *V*_*oc*_ is proportional to the applied stress σ_*xy*_ and the gap distance between electrodes *g*_*e*_.^10^ Since the piezoelectric film layer is normally very thin, an IDE structure has the advantage of generating enhanced output voltage due to the higher gap distance between electrodes in the IDE structure than that in the MIM structure.^10,136^Figure 10c,d are the structure of bimorph cantilever in series and parallel, respectively. There is a shim between the two separated piezoelectric sheets. When the bimorph cantilever is subjected to external force, the top and bottom piezoelectric sheets are in different mechanical status: tension/compression or compression/tension, respectively.^10^ According to the different electrode connections, the bimorph cantilever can induce accumulated current or voltage through the two layers in series or parallel.

In 2006, Wang’s group first reported a ZnO nanorod-based piezoelectric nanogenerator by using an atomic force microscope (AFM) to strain the NRs individually, generating around 8 mV and 10 pW/μm^2^ from one nanorod.^34^ As shown in Figure 10e,f, a Pt-coated AFM tip was used to apply a force of 5 nN to deflect the ZnO NRs causing the outer surface of the ZnO NRs to stretch under positive strain and the inner surface compressed under negative strain.^34^ It was postulated that a Schottky barrier formed between Pt (φ = 6.1 eV) and n-type ZnO (electron affinity of ZnO is 4.5 eV) could cause the accumulation of net charge formed and drive the electrons flowing from the ZnO to metal.^138^ Then, the flow of electrons from the external circuit will neutralize the ionic charges causing a measurable output voltage.^34^ When the ionic charges are fully neutralized, the output voltage drops to zero. Thus, in this work, it was demonstrated that a Schottky junction formed between the contact is the key factor for the piezoelectric nanogenerator to create a charge accumulation and releasing process leading to a current flowing from the electrode to the ZnO NRs. Following this initial work, in order to actuate all the signal from ZnO NRs simultaneously and continuously, Wang’s group used a zigzag metal electrode on the top of ZnO NRs array and ultrasonic wave to drive the device shown in Figure 10g.^137^ The ultrasonic wave could apply continuous stretching and compressive forces on the NRs by using zigzag metal electrode, which generated output voltage and current around 0.7 mV and 0.15 nA. The zigzag electrode acted like an array of AFM tips applying mechanical force on the ZnO NRs to achieve continuous energy harvesting. The above research led to a significant increase in the study of nanostructured piezoelectric materials for energy harvesting devices. Different strategies to improve the performance of the energy harvesters were investigated, which will be discussed in sections 4.2 and 5.

### 4.2 Solutions to Screening Effect

When a force is applied to produce piezoelectric potential in piezoelectric materials, an electric field will be created due to the polarization, known as the depolarization field.^139,140^ Under this condition, the electric field induces carriers from the piezoelectric material and contacts to move to screen the electrical field to zero, called internal and external screening, respectively. The internal screening is mainly due to the free carriers within piezoelectric materials.^141^ It is well-known that ZnO, BaTiO_3_, and MoS_2_ have a high density of surface-induced free carries due to the vacancies and impurities (hydroxide (OH−) groups) on the surface, which also strongly affects their conductivity.^142^ The external screening is mainly from the metal electrode due to the high density and mobility of carriers at the metal surface.^140^ Because the depolarization potential can be screened completely after a given time, the rate of screening determines the ability to measure a voltage and transfer charge to an external circuit.

In general, the screening effect adversely affect the output performance of energy harvesters. Therefore, reducing the screening rate is important to generate a voltage or develop a higher voltage. It has been reported that the rate of screening effect can be reduced through decreasing the density of surface-induced free carriers in the piezoelectric material or creating a depletion region between the piezoelectric material and contacts. There are various approaches to reduce the screening effect.

#### 4.2.1 Chemical Doping

The piezoelectric constant can be modified to enhance the performance by chemical doping. According to different piezoelectric materials, there are various chemical dopants to optimize their properties. The original crystal lattice’s atoms in the lattice can be replaced by incorporating atoms by substitution or interstitials, leading to structural deformations. In terms of the material structure, they can also generate tensile strain affecting the lattice constant to synergistically modify the piezoelectric properties of material to obtain higher performance. The dopants can be classified into two categories: p-type and n-type. ZnO is often an n-type semiconductive material. Generally, p-type dopants were used to modified ZnO to reduce the free electron density, as a consequence, lower the screening effect. In the case of n-type doping, n-type doping can reduce crystal lattice strain to effect the piezoelectric coefficient to improve the output performance.^36,143^ As shown in Table 2, metal and halogen ions were demonstrated as dopants for ZnO to improve output voltage and current and its corresponding peak to peak voltage (*V*_pp_) and current (*I*_pp_). By controlling the morphology and concentration of the chemical dopants, they exhibit obviously higher performance after doping, which can be regarded a promising route toward boosting the performance of piezoelectric energy harvesters. Sometimes, chemical doping also suffers from poor stability of the doped materials as the result of the formation of low energy donor impurities such as hydrogen interstitials and oxygen vacancies.^6^

**Table 2:** Output Performance of Doped Piezoelectric Generator

		output voltage		output current	
samples	morphology	undoped	doped	undoped	doped
Br-doped ZnO^144^	NRs	2.4 V (*V*_pp_)	5.90 V (*V*_pp_)	400 nA/cm^2^ (*I*_pp_)	1910 nA/cm^2^ (*I*_pp_)
Br-doped ZnO^145^	nanosheets (NSs)	5.9 V	8.82 V	2.95 μA/cm^2^	8.89 μA/cm^2^
Cu-doped ZnO^146^	NSs	10 mV	35 mV	7–8 nA	50 nA
Ni-doped ZnO^147^	NRs	0.006 V	0.07 V	0.0733 μA	10.5 μA
Tb-doped ZnO^148^	NRs	2.3 V	9.0 V		
La-doped ZnO^149^	NRs	2.1 V	3.0 V		
Li-doped ZnO^150^	NRs		160 mV	1.6 nA	8 nA
Ag-doped ZnO^151^	NRs	2.28 V (*V*_pp_)	6.85 V (*V*_pp_)	1.16 μA (*I*_pp_)	3.42 μA (*I*_pp_)

#### 4.2.2 Surface Treatment

Intrinsic oxygen vacancies within the piezoelectric materials are a major fundamental source of free-carriers. Previous works reported that oxygen vacancies could be reduced by using oxygen plasma treatment.^152^ The major species, O*, can react with the surface-adsorbed H atoms and fill oxygen vacancies because oxygen plasma comprises a variety of oxygen ions and radicals such as O^+^ and O^2+^.^152^ Hussain et al. found that the average output potential from plasma-treated ZnO NRs by using an AFM tip increased from 78 to 122.7 mV due to the decreased free carrier concentration.^152^ However, it showed that since H atoms might readsorb on the surface of ZnO or plasma-induced O* injection could effuse, the impact of plasma treatment on the surface of materials is probably not very stable under air circumstances.^1,153^ Annealing under different environments is an alternative method to help reduce the intrinsic defects and contaminants in ZnO NRs. It has been reported that the oxygen vacancies could be decreased by annealing in air above 200 °C, while −OH groups at the surface of ZnO could be significantly reduced by annealing above the temperatures of 150 °C.^154^ Hu et al. reported the output voltage and current improve from 5 to 8 V and 300 nA to 900 nA after annealing the ZnO at 350 °C.^153^ They also compared the performance of the annealing-treated energy harvester after 1 month and oxygen-plasma-treated energy harvester after 2 weeks. There was no obvious performance decrease of the device treated by annealing measured after one month, while the performance of the device treated by oxygen plasma decreased after 2 weeks indicating that annealing treatment is more stable compared to oxygen-plasma treatment. However, annealing treatment generally needs high temperature (>200 °C), which is only applicable for some rigid substrates;^1^ flexible and polymer substrates generally cannot be treated at temperatures higher than ∼150 °C. Immersing materials into an insulating layer to make the surface more chemically inert can be used to protect the device from electrical leakages through the internal piezoelectric materials and short circuits that could occur during the measurement.^155^ PMMA is the most common used polymer, which can fill the gaps between NRs to increase mechanical robustness and prevent electrical shorts.^156^ It has been reported that the power density can be improved 20 times by the synergistic effect of oxygen plasma, annealing treatment, and PMMA surface passivation on ZnO NRs.^153^ The insulation layer can improve the mechanical robustness and reduce the effect of capacitance changes that may occur during the mechanical pressing and releasing actions. Parylene C polymer was also used as insulating layer to enhance the piezo properties of ZnO with the maximum output voltage of 10 V.^157^

#### 4.2.3 Junction Effects

Interfacial modification to form a depletion region with an in-built electric field is another effective and stable technique to reduce carrier drift velocity and the screening rate, which could also isolate the surface of piezoelectric materials from atmospheric interactions. A Schottky junction can form between a semiconductor material with a wide bandgap and a metal with a large work function. At the early stage of research in the field,^34^ the Schottky contact formed at the metal–ZnO interface was considered to be an important energy barrier to improve the output performance of piezoelectric energy harvesters. The Schottky barrier can help accumulate the net charges at the interface area, as discussed in section 4.1. Then, the accumulated electrons will flow back when the mechanical deformation is released. It has been reported that Au NPs introduced on ZnO surface can form Schottky junctions to decrease the free carriers concentration with improved output performance by 10 times compared to the pristine ZnO nanogenerator.^158^ Some other metallic materials, such as Pd-,^159^ Ag-,^160^ and Ag-doped graphene,^161^ had been used to form Schottky junction with ZnO due to their unmatched work function. The Schottky junction will tend to drive the free carries in ZnO to this materials, resulting in a reduction in carrier density of ZnO to increase output of the piezoelectric energy harvesters.^36^

**Figure 11:**
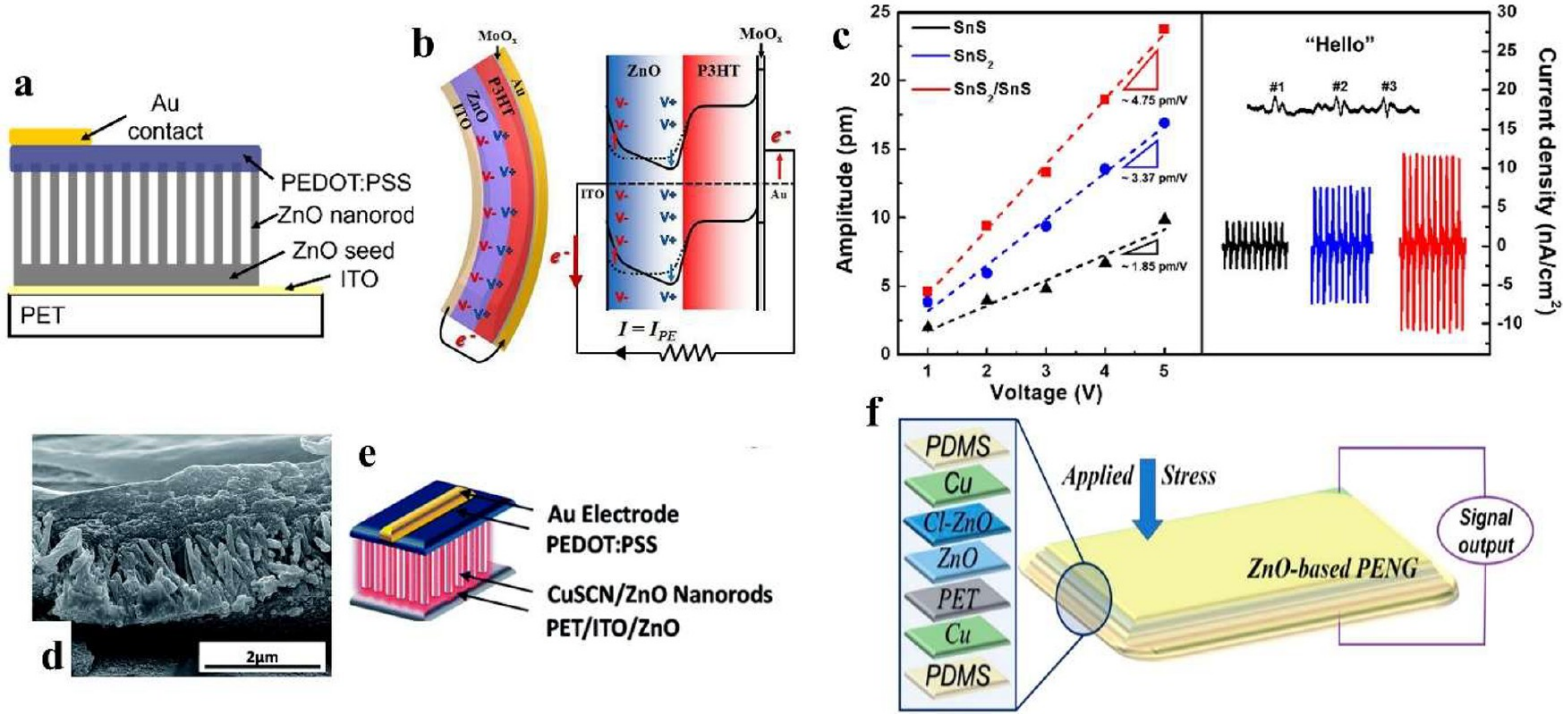
Piezoelectric energy harvesters with heterostructures giving junction effects. (a) Schematic of PEDOT:PSS/ZnO nanogenerator. Reproduced with permission from ref (166). Copyright 2012 John Wiley and Sons. (b) Schematic of P3HT/ZnO nanorod energy band. Reproduced with permission from ref (167). Copyright 2012 American Chemical Society. (c) Effective piezoelectric coefficient of SnS_2_/SnS heterostructure thin films and the device attached to the vocal cords tracing different words. Reproduced with permission from ref (169). Copyright 2021 American Chemical Society. (d) Cross-sectional SEM image of PEDOT:PSS/CuSCN/ZnO NRs and (e) the corresponding scheme of nanogenerator. Reproduced with permission from ref (170). Copyright 2014 Royal Society of Chemistry. (f) Schematic of CuO/Cl-doped ZnO nanogenerator. Reproduced with permission from ref (171). Copyright 2016 American Chemical Society.

As is well-known, p-type materials can be used to combine with n-type materials to create a depletion region by forming p–n junction between the interfaces. P-type polymer and small molecule semiconductors have been extensively researched in the field of organic electronics due to their excellent hole conductivity and mechanical properties such as poly(3,4-ethylenedioxythiophene) polystyrenesulfonate (PEDOT:PSS), poly(3-hexylthiophene) (P3HT), and Spiro-OMeTAD.^162−165^ These properties can be combined with piezoelectric materials to enhance overall performance. Figure 11a shows the structural schematic of the fabricated PEDOT:PSS on ZnO NRs nanogenerator, which can generate output voltage and current output in the ranges of 10 mV and 10 μA/cm^2^.^166^ Using the commonalities between piezoelectric and ferroelectric materials, the in-built electric field at the p–n junction can reduce electric field caused by the negative polarization at the ZnO/p-type material interfaces because of the reduced carrier drift velocity in the depletion region leading to decreased screening effect rate sufficiently for a voltage to be generated.^166^ The density and mobility of carriers in the semiconductor layer between the piezoelectric material and contact can help to reduce the rate of the screening effect as well.^166^ This suggests that a larger depletion region will slow the screening carriers even more, resulting in a greater output voltage. As shown in Figure 11b, P3HT was also investigated to form p–n junction with piezoelectric layer to develop higher performance. P3HT deposited on a ZnO layer can generate higher output voltage of 0.5 V compared to 0.08 V without P3HT at the strain of 0.068% because the free electrons in ZnO were passivated by attracting holes from P3HT further reducing the capacitance of the device.^167^ Spiro-MeOTAD as one of p-type semiconductor can be coated on ZnO nanodisks with ITO/PET as top and bottom electrodes show 300 nA output current at the vertical compressive force of 10 N, which is nearly 10 times higher than that of pristine ZnO control device.^168^

Due to the limitation of the high cost of some p-type polymers and potential unstable issues, some p-type oxide semiconductors were also investigated as alternative materials to fabricate p–n junction with piezoelectric materials to improve performance. Nie et al. used a cathodic deposition method to coat p-Cu_2_O on ZnO nanoarray to fabricate nanogenerator with 30 times enhanced output current of 900 nA compared to the ZnO NG without p-Cu_2_O layer. Some other p-type oxide semiconductors, such as NiO,^172^ CuO,^173^ CuI,^174^ and Sb-doped Cu_2_O,^175^ can be also synthesized via several possible methods such as high-vacuum, electrochemical deposition and high-temperature processes to form a p–n junction with piezoelectric materials to improve the performance. 2D heterostructure can also generate large band offset leading to large electric polarization and piezoelectricity such as WSe_2_/MoS_2_,^176^ In_2_Se_3_/MoS,^177^ and SnS_2_/SnS.^169^Figure 11c shows SnS_2_/SnS heterostructure thin films fabricated by atomic layer deposition exhibiting a piezoelectric coefficient of ∼4.75 pm/V with flexibility to be attached to vocal cords to trace different words.^169^ The generated charges from piezoelectric materials can be controllably transferred between the heterojunction/interface built due to the different band energies and relative positions.

The above solutions to reduce screening effect can be combined to synergistically enhance the performance of the device. The external screening effect and internal screening effect can be decreased by combining the formation of a depletion region at the material interfaces and reducing the free carriers from the piezoelectric materials leading to a decreased rate of polarization field screening to obtain higher device performance. Figure 11d-e shows ZnO NRs coated with p-type CuSCN to achieve surface passivation and deposited PEDOT:PSS as well to form p–n junction.^170^ Here, CuSCN can be considered as reducing the internal screening effect due to the surface induced mobile free carriers by a forming depletion region with ZnO, but also the chemical bond between CuSCN and ZnO to reduce the density of defects on ZnO surface to decrease carrier concentration. It was reported that the device with CuSCN coating showed a 5-fold increase in output voltage and current of 1.07 V and 1.88 mA/cm^2^ compared to the uncoated-CuSCN ZnO device.^170^ Polydiallyldimethylammonium chloride (PDADMAC) and polystyrenesulfonate (PSS) can also be deposited via layer by layer technique to surface modify ZnO NWs with enhanced output performance.^153^ PDADMAC:PSS-coated ZnO NRs for PEDOT:PSS/ZnO p–n junction energy harvesters presented an 8-fold output voltage of 1 V compared to the counterpart without PDDA:PSS.^178^ In addition, by combining chemical doping and p–n junction, CuO/Cl-doped ZnO nanogenerator shown in Figure 11f can obtain enhanced output voltage (*V*_pp_) and current (*I*_pp_) of ∼2.2 V and ∼1000 nA/cm^2^, respectively.^171^

## 5 Strategies to Improve Performance

### 5.1 Effect of Material Micromorphology on Performance

The various morphologies of identical materials significantly influence their piezoelectric characteristics and performance of the corresponding device. The micromorphologies of the materials can be controllably obtained by controlling the synthesis condition such as reaction temperature, pH value, template types, additives, and preparation process. There are various approaches to synthesize different materials such as ball milling, sol–gel approach, hydrothermal synthesis, molten salt reaction, CVD, mechanical and liquid exfoliation. Generally, the nanomorphology of the materials can be categorized into three categories, 0D materials (nanoparticle), 1D materials (nanowire, nanotube, nanorod and nanobelt), and 2D materials (nanoplate and nanosheet).

**Figure 12:**
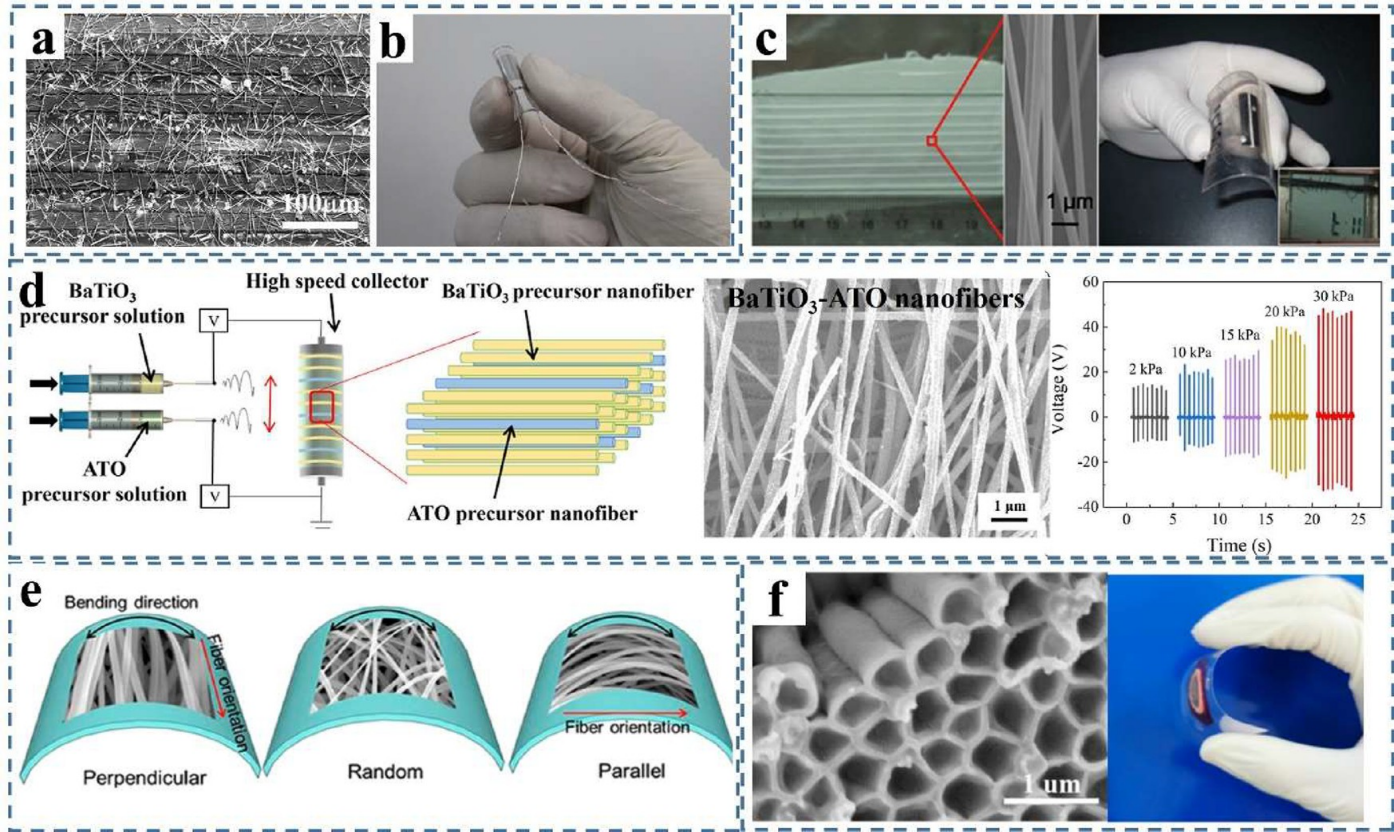
Different strategies for 1D inorganic piezoelectric material synthesis. (a, b) PZT NWs-based flexible transparency piezoelectric nanogenerator. Reproduced with permission from ref (179). Copyright 2017 American Chemical Society. (c) Aligned electrospun PZT nanofibers-based flexible energy harvester under bending. Reproduced with permission from ref (180). Copyright 2012 American Chemical Society. (d) BaTiO_3_-antimony-doped tin oxide (ATO) electrospun nanofibers and the scheme of electrospinning setup with ability to harvest energy from different pressure. Reproduced with permission from ref (181). Copyright 2022 American Chemical Society. (e) Flexible energy harvester fabricated by electrospinning PZT NWs with different orientation. Reproduced with permission from ref (182). Copyright 2019 American Chemical Society. (f) Free-standing PZT nanotube array and its flexible device. Reproduced with permission from ref (183). Copyright 2022 American Chemical Society.

It is important to design an energy harvester with the properties of the piezoelectric material well matched to the application. 1D materials with a small diameter are more sensitive to small applied force and vibration.^143,184^ Different morphologies and properties of 1D PZT were investigated to obtain PZT-based nanogenerator with high flexibility and efficiency. Kwon et al. patterned PZT ribbons by a typical photolithographic technique, followed by the etching of Pt/Ti/SiO_2_/Si multilayers.^185^ The whole PZT ribbons were subsequently stamped with polydimethylsiloxane (PDMS) and transferred to a PET flexible substrate with graphene sheets used as electrodes in place of a conventional metal to fabricate the energy harvester, which exhibited output voltage of 1.0 V, 1.5 and 2 V when subjected to a compressive force of 0.3 kgf, 0.6 kgf and 0.9 kgf, respectively. A liquid crystal display (LCD) was driven by the output voltage and current of 2 V and 2 μA/cm^2^. However, the process of fabricating PZT nanoribbons is complicated, which needed patterning and etching. Compared to PZT nanoribbons fabricated by a complex method with several steps, PZT NWs can be obtained by the hydrothermal method, which was used to form a PZT nanowire suspension to spin-coat on a mica sheet shown in Figure 12a,b.^179^ The PZT nanowire-based energy harvester exhibited increasing output voltage as the applied pressure increased from 15 to 70 kPa with maximum voltage and power density of 10 V and 0.27 μW/cm^2^ with 70 kPa applied pressure, respectively. Electrospinning is an alternative method to synthesize organic and inorganic materials, which can overcome the difficulties to grow larger-scale 1D single crystal NWs (above 50 μm). Generally, electrospinning needs a polymer-based precursor solution to help form the fibers. The fibers are extruded from the needle with a high voltage applied. Thus, 1D inorganic piezoelectric materials NWs can be obtained by mixing polymer solution and inorganic materials during electrospinning such as 0.5Ba(Zr_0.2_Ti0_.8_)O_3_–0.5(Ba_0.7_Ca_0.3_)TiO_3_ (BZT–BCT), PZT, and BaTiO_3_ NWs.^180−182,186^ The electrospun BZT-BCT fiber exhibits an output voltage of 3.25 V and output current of 55 nA under stretching.^186^ The grinding of BZT–BCT electrospun NWs can be further mixed with PVDF to obtain composited film and fibers by spin-coating and electrospinning used as piezoelectric nanogenerator, respectively.^187,188^Figure 12c shows synthesized PZT NWs by electrospinning deposited on a PET film to fabricate a flexible nanogenerator, which generated a 6 V output voltage by periodically bending and releasing.^180^Figure 12d shows the electrospinning setup for BaTiO_3_-antimony-doped tin oxide (ATO) nanofibers, where the high electrical conductive ATO can provide effective conductive paths to transfer the underlying charges generated from the internal BaTiO_3_ nanofibers to the surface area, inducing much more charges on the electrodes and yielding high outputs of 46 V and 14.5 μA at 30 kPa pressure.^181^ Electrospinning can also achieve aligned nanofibers by controlling the speed, electrical field, and the location of collector. The controlled orientation of electrospun PZT nanofibers was reported as well.^182^ As shown in Figure 12e, by using different direction of metal wires as collectors, the direction of PZT nanofibers can be changed because of the different electric field directions induced by the metal wires. The nanofiber mat was peeled off from the metal wire, then placed on ITO-PEN to fabricate a nanogenerator. The output performance is relied on the direction of bending and fiber orientation. When bending direction and fiber orientation were parallel, a large portion of the PZT nanofibers deformed longitudinally during bending leading to highest output performance. As show in Figure 12f, a PZT nanotube array was synthesized by NaOH etching anodic aluminum oxide (AAO) template, which can be fabricated as flexible device with sputtered Au and ITO electrodes demonstrating a higher flexoelectric coefficient 1.92 × 10^–9^ C/m by testing the current under tip displacement of the nanocomposite film by vibration.^183^

**Figure 13:**
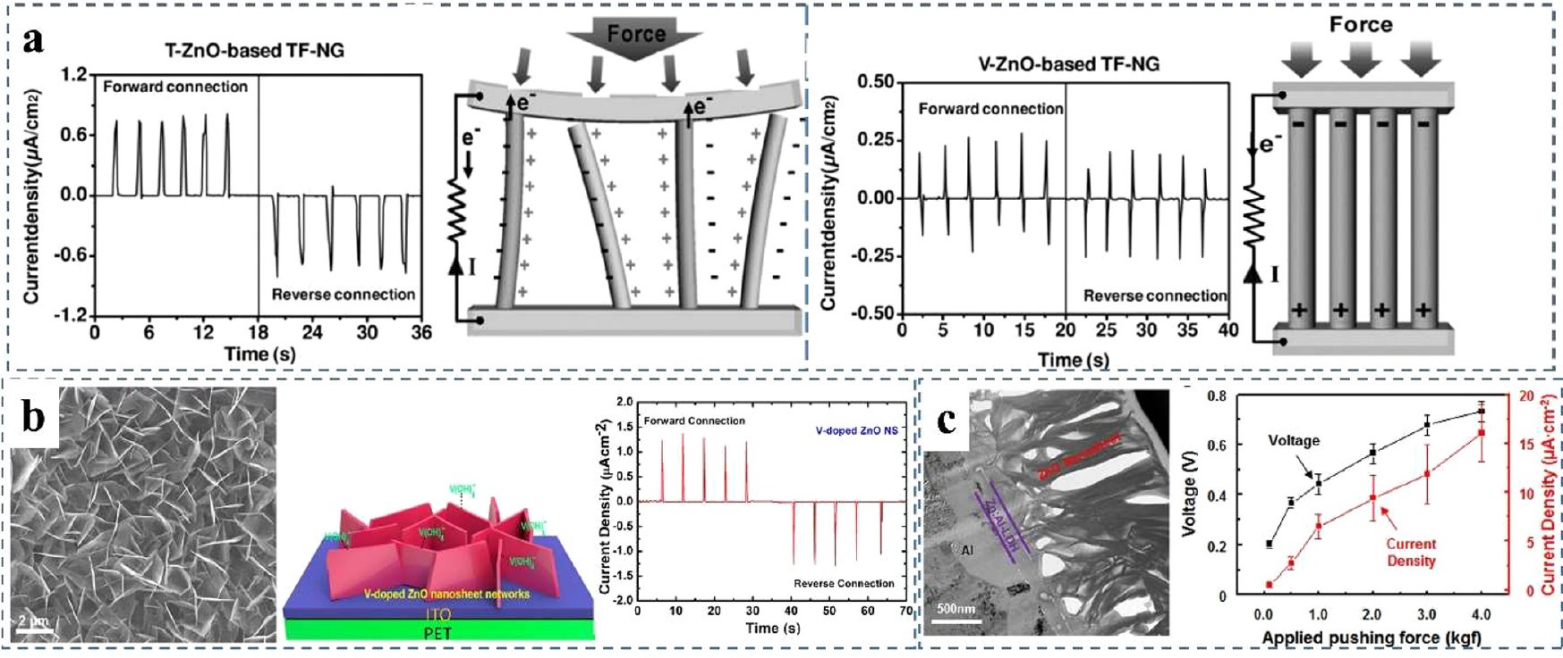
The influence of different ZnO morphologies on the performance. (a) Direct current (DC) and alternating current (AC) type output charges generation from titled (T) and vertical (V) ZnO NRs and the corresponding schematic, respectively. Reproduced with permission from ref (189). Copyright 2011 John Wiley and Sons. (b) SEM image of ZnO NSs grown on ITO/PET and the schematic of NSs growth mechanism with DC output current under compression. Reproduced with permission from ref (190). Copyright 2013 American Chemical Society. (c) Cross-sectional TEM image of layered double hydroxide (LDH) as anionic layer at the interface between the ZnO NSs and the Al electrode and the output performance under different applied pushing force. Reproduced with permission from ref (191). Copyright 2013 The American Association for the Advancement of Science.

Moreover, the direction of ZnO NRs also influence the performance of the corresponding device. Tilted (T) and vertical (V) ZnO NRs shown in Figure 13a generated different power modes of direct current (DC) and alternating current (AC), respectively. As show in the schematic in Figure 13a, when a bending force was applied on tilted ZnO NRs, the stretched side of NRs showed positive potential, while the compressed side showed negative potential, which led to the polarization along the width of tilted ZnO NRs resulting in the DC-type output performance.^189^ For vertical ZnO NRs under compressing, it was explained that the vertical well-aligned ZnO NRs are more sensitive to compressive force in the direction of the nanorod length rather than being bent. When compressive force was applied on the vertical ZnO NRs, a piezoelectric potential was formed along the *c*-axis of the NRs, with negative piezoelectric potential on one side and positive piezoelectric potential on the other side leading to AC-type output signal.^189^ For most ZnO-based nanogenerators, ZnO NRs grown on a substrate combine the above two situations, thus the applied force on the device combining the above two situations causes an AC output performance.

2D NSs have attracted lots of attention due to their distinctive physical and chemical characteristics, high surface-to-volume ratio and mechanical durability. Kim’s group synthesized 2D vanadium (V)-doped ZnO NSs on ITO/PET by forming V(OH)_4_^–^ during the reaction to block the ZnO growth along the (0001) direction as shown in Figure 13b.^190^ It demonstrated that the backflow of accumulated electrons from the bottom electrode to the top electrode is prevented by V(OH)_4_^–^ on the top side of V-doped ZnO NSs, which displayed a DC voltage of 1.0 μA/cm^2^ under a vertical compressive force of 0.5 kgf. Then, as shown in Figure 13c, they investigated the influence of ZnO NSs/anionic layer heterojunction on the performance of ZnO-NSs based device, which generated DC voltages of around 2.5 and 6.5 μA/cm^2^ under 0.5 and 1 kgf pushing force, respectively. The ZnO-NSs based device with anionic layer heterojunction also exhibited 10 times larger strain-energy density of 8.2 × 10^7^ J/m^3^ under the same external mechanical loads compared to ZnO NRs with strain-energy density of 7.88 × 10^6^ J/m^3^.^191^ Here, a higher potential difference developed with the accumulation of negative charges at the anionic layer (LDH/Al electrode), driving electrons from the bottom side of the Al electrode to the top side of the Au electrode.^191^

0D (nanoparticle) and 3D piezoelectric materials (nanocube, nanoflower) also show good piezoelectric properties. However, due to their morphologies and synthesis methods, they generally have been combined with some polymer materials to form composite films to achieve energy harvesters with better performance, which will be discussed in the next section.

### 5.2 Development of Piezoelectric Composite Materials

With the development of wearable microelectronics, it is very desirable to develop a flexible device to harvest energy from various body movements. Therefore, some composite structures combining polymer and inorganic materials have attracted lots of attention to achieve better output energy with good flexibility and stability. The composite piezoelectric films are formed by mixing two or more parts of materials together without chemical reaction between them, which is regarded as a cost-effective and easy processing method to obtain energy harvesters with high performance. The polymer materials can act as supporting binder materials to hold the materials making the inorganic material a uniform dispersion within the composite material. In addition, the polymer materials can produce surface modification of the inorganic materials, reducing the internal leakage current and screening effect providing higher output power and good mechanical flexibility.

**Figure 14:**
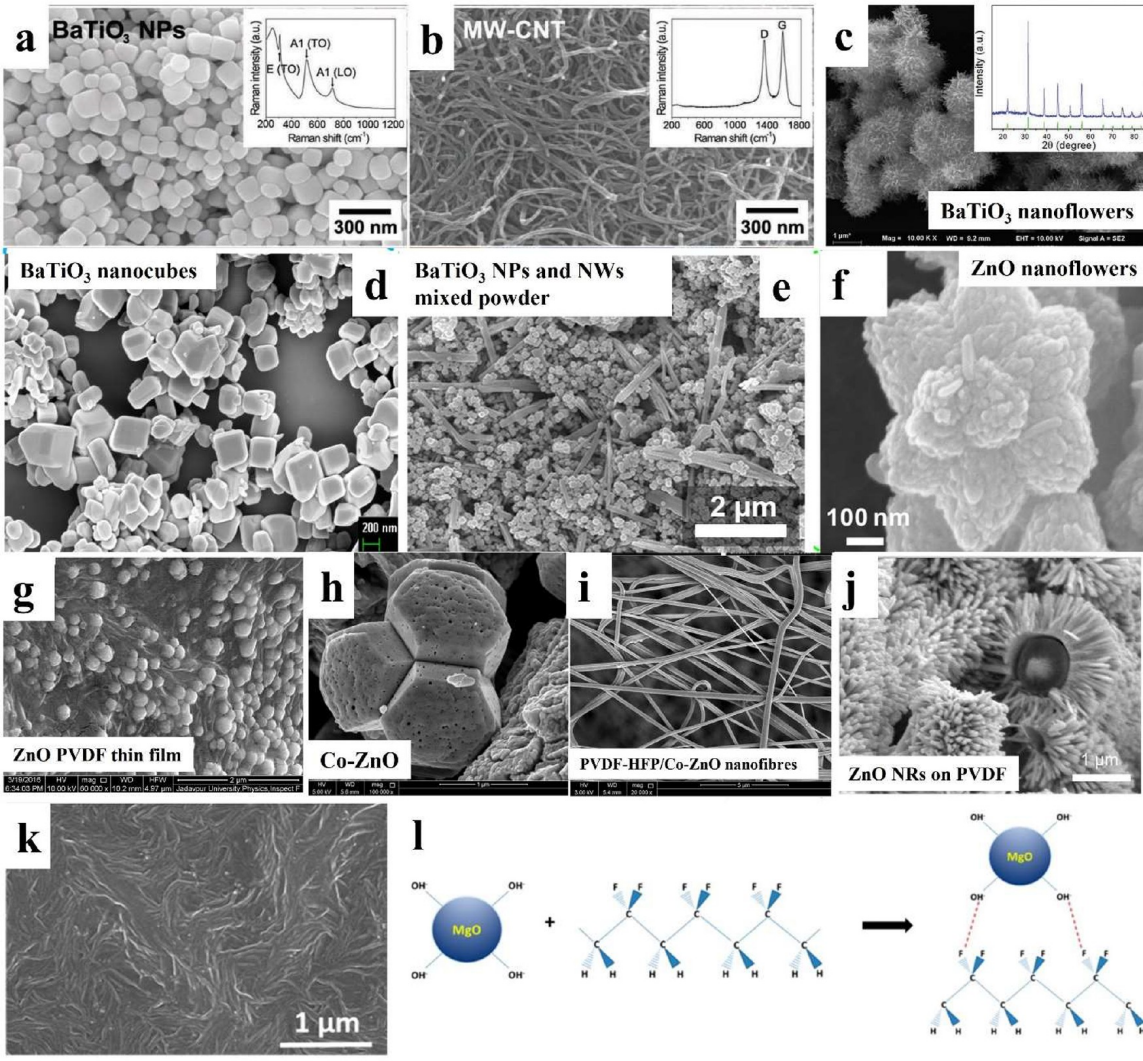
Inorganic materials with different morphologies used for composites materials synthesis with organic materials. SEM images of (a) BaTiO_3_ NPs and (b) muti-walled carbon nanotubes. Reproduced with permission from ref (192). Copyright 2012 John Wiley and Sons. (c) BaTiO_3_ nanoflowers. Reproduced with permission from ref (193). Copyright 2020 John Wiley and Sons. (d) BaTiO_3_ nanocubes. Reproduced with permission from ref (194). Copyright 2017 American Chemical Society. (e) Mixture of BaTiO_3_ NPs and NWs. Reproduced with permission from ref (195). Copyright 2014 Elsevier. (f) ZnO nanoflowers. Reproduced with permission from ref (196). Copyright 2018 American Chemical Society. (g) ZnO NPs/PVDF film. Reproduced with permission from ref (197). Copyright 2018 Elsevier. (h) Co-ZnO NPs and (i) PVDF-HFP/Co-ZnO electrospun nanofibers. Reproduced with permission from ref (198). Copyright 2018 Springer Nature. (j) ZnO NRs grown on PVDF electrospun fibers. Reproduced with permission from ref (199). Copyright 2020 Elsevier. (k) MgO/PVDF film. (l) Schematic of surface interaction between MgO and PVDF-TrFE. Reproduced with permission from ref (128). Copyright 2014 American Chemical Society.

PDMS with properties of very low stiffness from 800 kPa to 10 MPa (depending on the curing agent ratio and curing temperature) and ease of processing has been used as a matrix mixed with inorganic piezoelectric materials to form composite structures.^200^ Park et al. mixed BaTiO_3_ NPs and carbon nanotubes in PDMS to fabricate an energy harvester as shown in Figure 14a,b.^192^ It was reported that stress reinforcements could be enhanced via adding some nanoreinforcements such as carbon nanotubes and graphene NSs. Then, the output performance was also improved by increasing BaTiO_3_ concentration up to 40 wt % due to stronger interactions and higher effective surface area in the PDMS composite structure.^201^ Different morphologies of BaTiO_3_, such as nanocubes and a mixed structure of NPs and NWs, as shown in Figure 14c–e, have also been utilized to mix with PDMS to fabricate composite energy harvesters to investigate the output performance. The above composite film needed a high electric field to align the dipole orientation for better performance of piezoelectric BaTiO_3_-based devices. Table 3 summaries their output performance when subjected to different external mechanical forces. Some 3D ZnO nanoarchitectures were also used to fabricate composite materials. Figure 14f shows flower-like ZnO synthesized by a precipitation method used to fabricate piezoelectric and piezoelectric-assisted triboelectric hybrid nanogenerators with output voltages of 12.5 and 39.8 V, respectively.^196^

**Table 3:** Output Performance of Different Composite-Based Energy Harvesters

samples	matrix	output voltage	output current	applied forces
BaTiO_3_ NPs/carbon nanotubes^192^	PDMS	3.2 V	350 nA	bending
BaTiO_3_ NWs/NPs^195^	PDMS	60 V	1.1 μA	5 mm bending at a rate of 0.2 m/s
BaTiO_3_ Nanocubes^194^	PDMS	126.3 V (*V*_pp_)	77.6 μA/cm^2^	pressure of 988.2 Pa
BaTiO_3_ Nanoflowers/carbon nanotubes^193^	PDMS	260 V	50 μA	compress force of 50 N and at 3.5 Hz
0.6 wt % ZnO nanoflowers^196^	PDMS	12.5 V	0.48 μA	pushing forces of 16 N at 5 Hz
ZnO NRs^202^	PVDF	3.2 V	0.6 μA	pressing
ZnO NPs^197^	PVDF	24.5 V	1.7 μA	pressure of ∼28 N at ∼5 Hz
Co-doped ZnO NPs^198^	PVDF-HFP	2.8 V		vibrating at 50 Hz
MgO NPs^128^	PVDF-TrFE	2 V		6 mm bending at 1 Hz
SnO_2_ nanosheet^203^	PVDF	42 V	6.25 μA/cm^2^	biomechanical stress of ∼0.3 MPa
PZT ceramic powder^204^	PVDF	55 V		Finger pressure of ∼8.5 KPa
BNT-ST ceramic powder^205^	PVDF	1.31 V (*V*_pp_)		Ultrasonic frequency of 6 MHz
BaTi_2_O_5_ NRs^206^	PVDF	53.2 V (*V*_pp_)		Vibrating under 3 g acceleration at 13 Hz
BaTiO_3_ NWs^207^	PVDF-TrFE	14 V	4 μA	bending
BaTiO_3_ NPs/carbon nanotubes^208^	PVDF	19.3 V	415 nA	impacting at acceleration of 5 m/s^2^
Ce–Fe_2_O_3_ NPs^209^	PVDF	20 V (*V*_pp_)	0.01 μA/cm^2^	2.5 N
Ce–Co_3_O_4_ nanocubes^209^	PVDF	15 V (*V*_pp_)	0.005 μA/cm^2^	2.5 N

However, polymer materials such as PDMS and PMMA can only be used as a passive support layer because they are nonpiezoelectric materials, which cannot provide extra piezoelectric signal. It is expected that organic polymer materials, including PVDF and its copolymers (PVDF-TrFE and PVDF-HFP), can be used as a piezoelectric active layer because their own electro-active β-phase can contribute to converting mechanical energy into electrical signal, and provide support and surface modification layer to the inorganic materials at the same time. Lee et al. spin-coated PVDF on ZnO NRs grown on Au/Cr coated Kapton, which exhibited around 0.2 V output voltage and 10 nA/cm^2^ current density.^210^ It can be explained that the polar phase content in the ZnO/PVDF film is effectively increased by the ion–dipole interaction between the NRs and polymer chains due to the surface charge of NRs and – CH_2_ dipoles of the polymer matrix being negative and positive, respectively.^210,211^ ZnO NRs have also been directly grown inside and developed on a PVDF film by spin-casting PVDF-Zn(Ac)_2_ solution on a substate followed hydrothermal ZnO nanorod growth. The extension force of ZnO NRs grown inside the PVDF film can help draw the PVDF film to keep the β phase orientation due to the in situ orderly pulling effect of ZnO NRs,^212^ of which the device exhibited a maximum output of 3.2 V by finger pressing.^212^ Moreover, it was reported that the β-phase of PVDF and its copolymer could be improved by mixing nanosized inorganic materials through the surface interaction with these materials to stabilize conformation. The nanogenerator based on ZnO NPs/PVDF composite film shown in Figure 14g also exhibited high output voltage and current of 24.5 V and 1.7 μA.^197^Figure 14h,I shows electrospun PVDF-HFP/Co-doped ZnO composite nanofibers.^198^ Compared with the output voltage of 120 mV for a device with neat PVDF-HFP, PVDF-HFP with 2 wt % Co-doped ZnO exhibited 2.8 V enhanced output voltage. PVDF electrospun fibers can be used as a substrate to grow ZnO NRs by sputtering ZnO seed layer followed by hydrothermal synthesis as shown in Figure 14j, which can be used as flexible pressure and bending sensor with sensitivities of 3.12 mV/kPa and 16.89 V·mm, respectively.^199^ In addition, the inorganic additive does not have to be a piezoelectric material to enhance the performance. SnO_2_ NSs can be also mixed with PVDF as piezoelectric energy harvester to get a maximum voltage of 42 V and current density of 6.25 μA/cm^2^ as shown in Table 3. It can be explained that the surface charges on these inorganic materials can interact with the molecular dipoles (CH_2_ or CF_2_) of PVDF and its copolymers to improve the β-phase content of the composites. Similarly, Singh et al. reported MgO NPs were embedded in PVDF-TrFE.^202^Figure 14k shows MgO/PVDF-TrFE composites film, which were cast on to an ITO-coated PET film to produce an energy harvester. The piezoelectric coefficient of PVDF-TrFE with 2 wt % MgO showed a 50% increase as compared to pure PVDF-TrFE, which can be attributed to the weak van der Waals forces between −OH groups on the MgO surface and F atoms in the PVDF-TrFE chains enhancing crystallinity as shown in Figure 14l.^128^ It was reported that surface-modifying n-type graphene (n-Gr) on a PVDF-BaTiO_3_ nanocomposite free-standing film could also help align the dipoles in one direction due to the majority of negative charge carriers of n-Gr, which exhibited a maximum output voltage (*V*_pp_) of 10 V along with a current (*I*_pp_) of 2.5 A at an applied force of 2 N.^213^ As shown in Table 3, some other inorganic materials including PZT, BNT-ST (0.78 Bi_0.5_Na_0.5_TiO_3_–0.22SrTiO_3_) ceramic powder, BaTi_2_O_5_ NRs, and BaTiO_3_ NWs and NPs, can be also used to form composite materials with PVDF exhibiting good output performance. The performance of composite-based energy harvesters can be further improved by optimizing the concentration of the nanofillers in the polymer matrix,^201,203^ electric dipole orientation,^212,213^ interfacial interaction,^128,209,214^ and synergistic effect between the inorganic nanofillers with polymer matrix.^214,215^

### 5.3 Special Top Electrode Design

**Figure 15:**
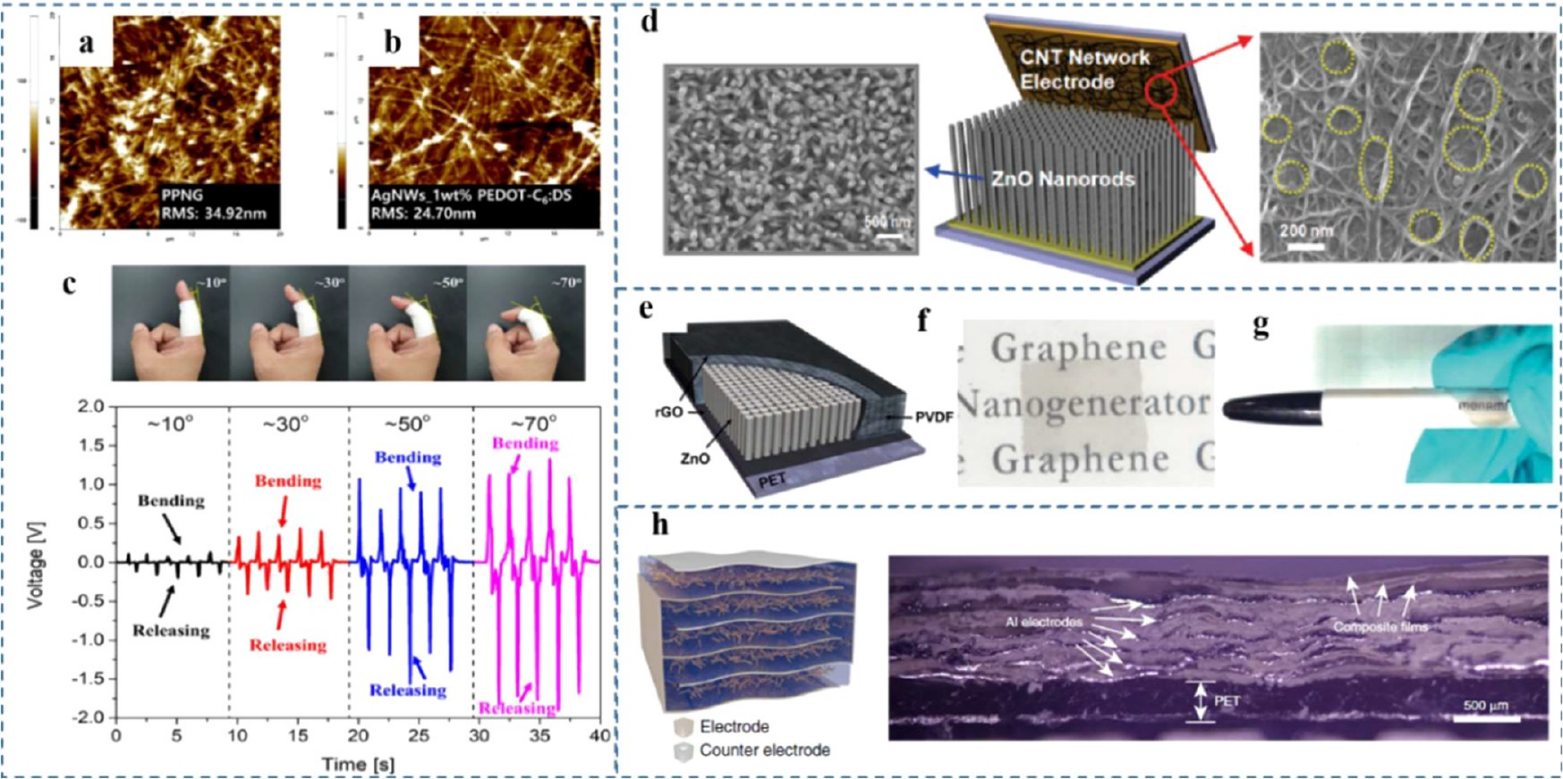
Special electrodes used for energy harvesters. (a, b) Surface roughness of Ag NWs and PEDOT-C6:DS coated Ag NWs as top and bottom electrode on PVDF film. (c) Output voltage of device under different finger bending degree. Reproduced with permission from ref (220). Copyright 2019 Elsevier. (d) Schematic illustration of an integrated nanogenerator with carbon nanotube as the top electrode. Reproduced with permission from ref (219). Copyright 2010 American Chemical Society. (e) Schematic of rGO electrodes for ZnO/PVDF composite film.^221^ Copyright 2015, Springer Nature. (f, g) Picture of CVD synthesized graphene on ZnO NRs as integrated fully rollable graphene-based nanogenerator. Reproduced with permission from ref (222). Copyright 2010 John Wiley and Sons. (h) Schematic diagram of nanogenerator with Al intercalated electrodes and cross-section image of fabricated nanogenerator with Al electrodes, composite films and PET are marked by white arrows. Reproduced with permission from ref (223). Copyright 2020 Springer Nature.

Generally, metal electrodes have been used as the top electrode on the active piezoelectric material layer such as Au,^170^ Ag,^216^ Cr/Au,^153^ Al,^217^ and Pt,^218^ which can be deposited by various methods such as sputtering and evaporation. However, the flexibility of the top electrode will affect the mechanical characteristics and endurance of the devices for wearable energy harvesting applications in the future. In addition, the rough surface can lead to low contact between the electrode and active layer during pushing or bending, especially for nanorod-based energy harvesters resulting in lower output performance.^219^ Ag NWs have gained lots of attention because of their high conductivity, low sheet resistance and excellent mechanical flexibility. As shown in Figure 15a,b, poly(2-hexyl-2,3-dihydrothieno[3,4-*b*][1,4]dioxine:dodecyl sulfate (PEDOT-C6:DS) can be deposited on Ag NWs used as top and bottom electrodes on PVDF film, which can reduce the surface roughness of Ag NWs from 34.92 to 24.7 nm providing good conduction pathways for charge transport and collection.^220^ It displayed maximum output voltage and current of 7.02 V and 1.11 μA and can be operated by finger bending from 10 to 70° as shown in Figure 15c.^220^

Some substrate-like electrodes including ITO/PET, Al foil, silver tape, etc., can be also used as a top electrode, especially for the fabrication of free-standing energy harvesters.^212,224,225^ Therefore, some top electrodes with special designs or using other conductive materials have been fabricated to enhance stability, mechanical properties and the output performance. As shown in Figure 15d, a carbon nanotube (CNT) film has been used as the top electrode on ZnO NRs providing high contact probability between ZnO NRs ascribed to CNT film with a networked surface topology and large pores resulting in enhanced output current density of 4.76 μA/cm^2^ at a pushing force of 0.9 kgf.^219^ However, the significant roughness of CNT also hindered the performance and development of further wearable applications.^226^ Graphene, as a 2D material, has attracted a lot of attention due to its chemical stability, electrical conductivity and high mechanical elasticity (elastic modulus of about 1 TPa).^227^ It can be prepared via various methods such as mechanical exfoliation and ultrasonic cleavage of graphite.^222,228−230^ As shown in Figure 15e, reduced graphene oxide (rGO) was used as bottom and top electrodes for PVDF-coated ZnO NRs by reduction of an inkjet-printed graphene oxide (GO) layer with vacuum assistance, which was able to detect variations in temperature between 20 and 120 °C and pressure fluctuations as tiny as 10 Pa.^221^ Large-scale graphene sheets with high-quality optical and electrical properties were obtained by using CVD as shown in Figure 15f,g, which could also be used as the top and bottom electrode of ZnO NRs to fabricate a flexible nangenerator rolled around a pen exhibiting output current density of 2 mA/cm^2^.^222^ MXene (Ti_3_C_2_) can be also used as a stress-match electrode for energy harvester because its conductivity is almost unaffected by mechanical deformations, even bending or twisting, which effectively resolves the short circuit issue with conventional electrodes. Flexible MXene (Ti_3_C_2_) electrodes were spray coated on to an electrospun PVDF/ZnO nanofiber mat as top and bottom electrodes, which exhibited bending sensitivity of 4.4 mV/deg under large range of bending degree from 44 to 122° due to the synergistic effect of PVDF/ZnO and flexible polymer electrode.^231^

In addition to using organic conductive materials as top electrodes, the design structure of the electrode also affects the performance of a nanogenerator. Intercalated electrodes have been used to improve the output power by connecting multiple piezoelectric layers in parallel or series. Gu et al. designed a kind of device with PDMS-coated Al intercalated electrodes on PVDF film as shown in Figure 15h. The output current and voltage reached 329 μA and 28 V under stress of 0.1 MPa.^223^ The intercalated electrode can add up the current generated from each unit to the large output current to get higher output current and voltage.^223,232^ Jin et al. designed an Ag intercalated electrode between PVDF layer as well, which generated 70 times larger piezoelectric power than a normal top/bottom electrode configuration.^232^

## 6 Wearable Piezoelectric Generators

With the increasing development of portable and biomedical devices, it is expected that portable electronics will be integrated into people’s clothes for using in the fields of medicine, entertainment, monitoring, healthcare, etc. However, traditional rechargeable batteries face limitations in energy storage capacity and large volume, leading to interruption of the monitoring process during recharging. Therefore, wearable energy harvesters attract lots of attention because they can convert energy from everyday body movement such as moving a finger, knee, or elbow and foot stepping into electrical signals to power the microelectronics and achieve continuous energy harvesting and powering. In addition, energy harvesters can prevent the problem of leakage of electrolyte solution and explosion from traditional rechargeable batteries. Therefore, it is expected that integrating piezoelectric materials into fiber or fabric substrates to fabricate energy harvesters with good flexibility and comfort can achieve high sensitivity, reliable energy conversion, and good mechanical properties at same time. Herein, we will discuss the wearable piezoelectric generator fabricated by using fiber or fabric substrates.

### 6.1 Fiber-Based Energy Harvesters

**Figure 16:**
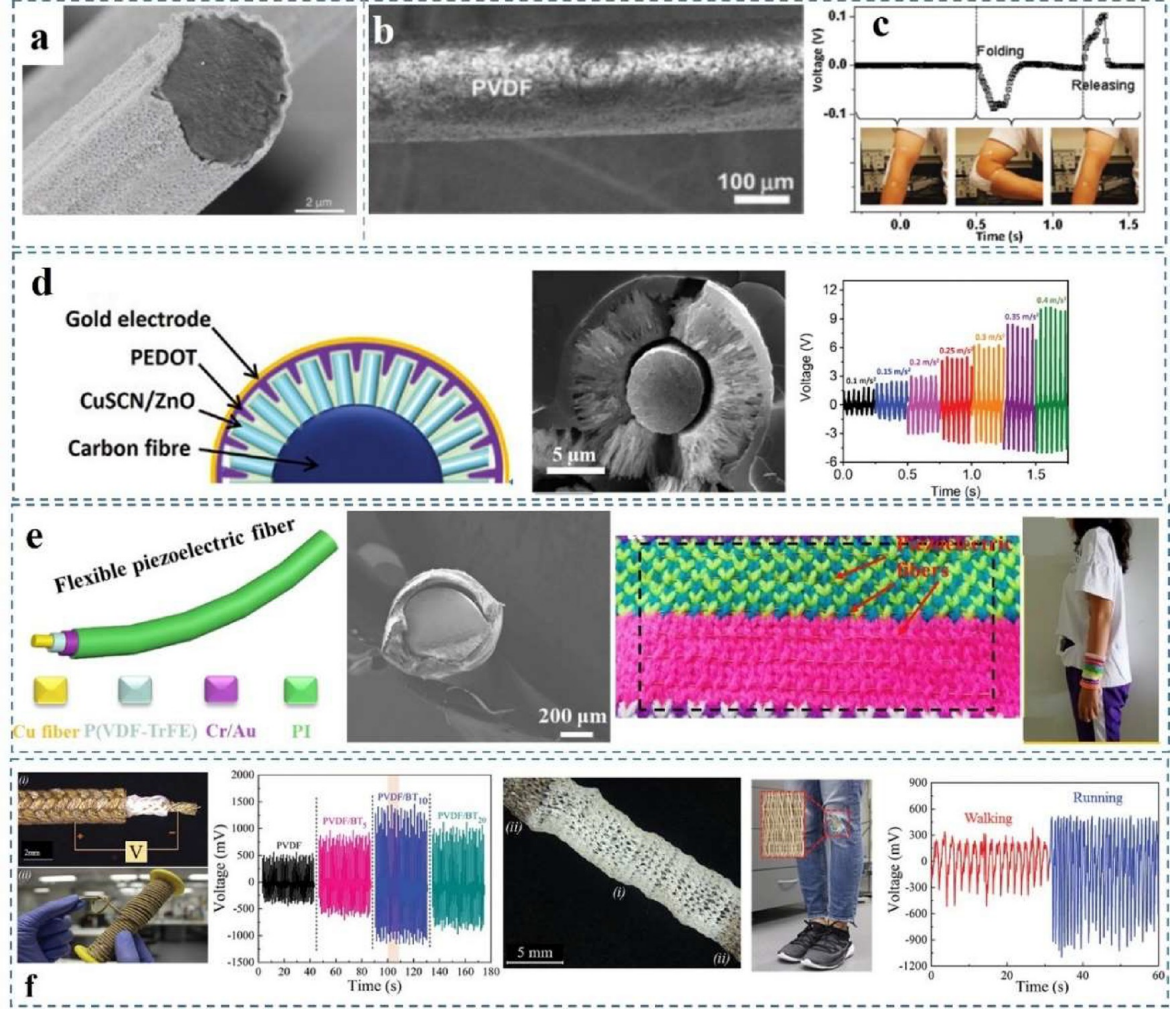
Energy harvester fabrication based on fibers. (a) SEM image of ZnO NRs grown on carbon fiber. Reproduced with permission from ref (236). Copyright 2011 John Wiley and Sons. (b, c) SEM image of PVDF/ZnO NRs on carbon fibers device with ability to harvest energy from arm bending. Reproduced with permission from ref (210). Copyright 2010 John Wiley and Sons. (d) Scheme and SEM image of PEDOT/CuSCN/ZnO carbon fiber-based devices with different output voltage at different impact acceleration. Reproduced with permission from ref (240). Copyright 2023 John Wiley and Sons. (e) Core–shell structure of PVDF-TrFE electrospun nanofibers on Cu wires as inner electrode stitched into fabric was used as self-power sensor. Reproduced with permission from ref (242). Copyright 2020 Elsevier. (f) Photograph of tri-axle braided piezo generator with the inner and outer silver-coated nylon electrodes, while the middle layer is made of braided PVDF/BaTiO_3_ fibers, which can harvest energy from compressing and is knittable as textile sewed on cloth to monitor walking and running. Reproduced with permission from ref (247). Copyright 2020 John Wiley and Sons.

Yarn- or fiber-like energy harvesters have several advantages compared to film-like energy harvesters, including light weight, enhanced interaction surface, and bendability. Additionally, yarn-like materials can be integrated into various desired shapes such as wearable textiles by weaving, knitting, sewing, or embroidering fabrics.^233,234^ Generally, fiber-based energy harvesters need coaxial or core–shell structures as electrodes used for collecting piezoelectric-polarization-induced current. Carbon fibers, as one good candidate, have gained great interest because of lightweight, high flexibility and good electrical properties, which can also be woven to make in desired wearable devices. Some researchers reported ZnO NRs grown on carbon fibers by the hydrothermal method for flexible UV detector and sensor to determinate dopamine concentration.^235^ Li et al. synthesized ZnO NRs on carbon fibers shown in Figure 16a by a physical vapor deposition method,^236^ which can generate maximum output voltage and average current density from the device was 3.2 V and 0.15 μA/cm^2^, respectively under air pressure driven inside a syringe. It was also reported that ZnO grown on carbon fiber can enhance mechanical performance by increasing the interfacial strength.^237^Figure 16b shows that the hybrid-fiber nanogenerator made of PVDF and ZnO NRs on Au-coated fiber was attached on a human arm to harvest energy from bending with output voltage and current density of 0.1 V and 10 nA/cm^2^, respectively, as shown in Figure 16c.^210^ Here, PVDF could be used as surface treatment on ZnO NRs to make the surface chemically inert, and the negative charge on ZnO surface also helped the piezoelectric dipole alignment in PVDF as discussed in section 5.2.

In the above situation, nanogenerators made from ZnO NRs on carbon fibers need acid etching of the as-grown ZnO NRs at one end of the carbon fibers to expose bare carbon fiber as one electrode. The counter electrode can be deposited on ZnO NRs such as Au, ITO/PET, and Ag. However, the etching process is complicated and can easily damage the electrode deteriorating the output performance and durability of the energy harvesters. To avoid the etching process of electrode deposition, Liao et al. used ZnO NRs on carbon fibers as bottom electrode and Au-coated ZnO NRs on paper as top electrode to fabricate a multifiber nanogenerator.^238^ A 200-carbon-fiber-based nanogenerator exhibited 100-fold improved output current of 35 nA. In addition, a separated electrode, such as Ti/Cu double side coated nylon film, could be used as the top electrode to wrap around ZnO NRs grown on carbon fiber to fabricate piezoelectric nanogenerator and triboelectric nanogenerator with output powers of 10.2 and 42.6 mW/m^2^, respectively.^239^ The fiber-based structure could be woven together to increase the generated output power through the synergy of the piezoelectric and triboelectric effect. However, for the device with a separated top electrode, the separated electrode may affect the flexibility and durability of the device, restricting the applicability of such fiber-based energy harvesters. Then, as shown in Figure 16d, PEDOT/CuSCN/ZnO was fabricated on carbon fibers, where the PEDOT can form a planar surface on ZnO nanorod to aid gold evaporation as a top electrode. The device could be used as self-powered sensor to identify different impact accelerations, which can be further designed into impact sensing board to identify impact location.^240^

Polymer fibers also have great advantages for energy harvesting due to the good flexibility, stretchability and mechanical stability. PVDF fibers can be prepared via continuous melt extrusion such as electrospinning, touch spinning, wet spinning, and melting spinning,^241^ which can be fabricated into energy harvesters by using a prespun nanofiber mat or weaving fibers together. PVDF-TrFE can be also electrospun on conductive wires/yarns to form core–shell structure with the inner conductive wire used as electrode, the deposited metal on PVDF-TrFE as the outer electrode. As shown in Figure 16e, the electrospun PVDF-TrFE on Cu wire formed a core–shell structure with sputtered Au/Cr as top electrode, which was stitched with textile as wearable sensor exhibiting 60.82 mV/N pressure sensitivity.^242^ Conductive nylon yarn was also used as inner electrode with electrospun PVDF nanofibers wrapped around, of which the outer electrode was evaporated silver on the PVDF with the ability of generating an output voltage and current of 0.52 V and 18.76 nA under cyclic compression of 0.02 MPa at 1.85 Hz with good durability.^243^ Some nanofillers such as antimony sulfoiodide NWs and BNT-ST NRs have also been added into PVDF and its copolymer PVDF-TrFE to fabricate hybrid yarns as energy harvesters exhibiting performance under different mechanical force.^241,244,245^ Mokhtari et al. braided melt-spun PVDF piezoelectric fibers and conductive silver coated nylon yarns together by using a Trenz–Export braiding machine with the conductive silver-coated nylon yarns as inner and outer electrodes.^246^ The braided PVDF-fiber nanogenerator showed an output voltage of 380 mV under compression and bending with high durability enduring 50% strain for thousands of bending cycles. Then, as shown in Figure 16f, they also fabricated a PVDF/BaTiO_3_ fiber nanogenerator by using same braiding method to harvest energy from compression. The PVDF/BaTiO_3_ braided fiber can be further knitted as a textile, which can be integrated with cloth as a wearable nanogenerator to monitor walking and running.^247^

### 6.2 Textile-Based Energy Harvesters

Fiber-woven textile such as cotton, polyester and acrylonitrile, hold enormous promise for wide-ranging applications in the future for wearable electronic devices, sensors and health monitoring systems due to their excellent lightweight, comfort and mechanical durability.^248^ Moreover, the porous structure and surface area of these textiles can afford more active sites and warrant easier passage of the electroactive materials resulting in a higher areal power and energy density. Additionally, some yarn-like materials can be further woven into a fabric textile energy harvester.

**Figure 17:**
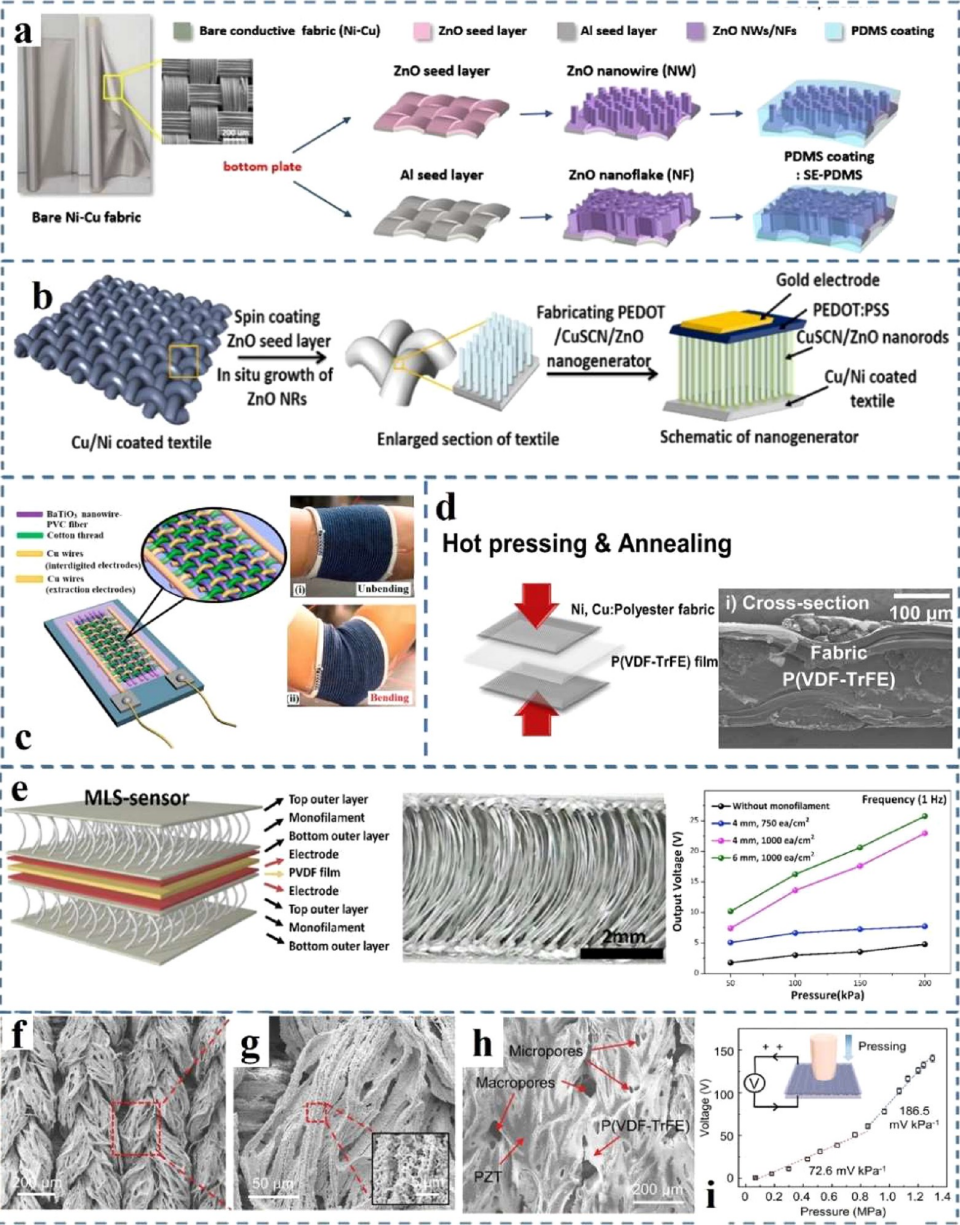
Energy harvester fabrication based on textile/textile pattern. (a) ZnO NWs and nanoflakes on Ni–Cu fabric energy harvesters fabrication scheme. Reproduced with permission from ref (252). Copyright 2018 American Chemical Society. (b) Textile-based PEDOT:PSS/CuSCN/ZnO piezoelectric nanogenerator fabrication scheme. Reproduced with permission from ref (254). Copyright 2021 Elsevier. (c) Fabric nanogenerator based on woven PVC/BaTiO_3_ fibers scheme. The inset pictures show device attached on arm. Reproduced with permission from ref (255). Copyright 2015 Elsevier. (d) Fabric-PVDF-TrFE heterostructure scheme via hot pressing and annealing and its cross-sectional SEM. Reproduced with permission from ref (256). Copyright 2020 Elsevier. (e) Multilocal PVDF device scheme and its optical microscope cross-section image of 3D textile generating voltage under pressure from 50 Pa to 100 kPa. Reproduced with permission from ref (257). Copyright 2020 Elsevier. (f–i) PVDF-TrFE/PZT textile device. (f–h) SEM of PZT hierarchical structure enveloped by the PVDF-TrFE film (i) generating voltages under pressure from 0.08 to 1.3 MPa. Reproduced with permission from ref (258). Copyright 2021 John Wiley and Sons.

In early work, the piezoelectric performance of ZnO NRs grown on various textiles had been tested by AFM.^249−251^ Furthermore, ZnO with various morphologies, including NRs, nanoflakes, and Ag-doped ZnO, have been manufactured into energy harvesters on textiles as well.^252,253^ Choi et al. prepared Al-coated Ni/Cu fabric and ZnO nanoflakes on Al-coated fabric as top and bottom separated electrodes, respectively and fabricated triboelectric energy harvesters as shown in the schematic of Figure 17a.^252^ Doping technology can also be achieved on a textile-based substrate. Ag-doped ZnO were synthesized on Au-coated woven polyester fibers to increase performance for a sound-driven device with separated Au-coated woven counter electrode, of which the output power of 0.5 μW is 2.9 times higher compared to undoped ZnO NRs.^253^ As the separated top electrode may influence the flexibility and durability of the device, as shown in Figure 17b,^254^ the PEDOT:PSS layer not only can form p–n junction with ZnO NRs but also can help metal deposition to form a stable and conformal top electrode. The synergistic effect of p–n junction from PEDOT:PSS and surface passivation from CuSCN contributed to the textile-based nanogenerator generating increasing output voltage from 0.2 to 1.81 V as the shaking frequency increases from 19 to 26 Hz. with high stability and durability under 26000 cycles by shaking at 26 H.^254^

Furthermore, fiber- or yarn-like piezoelectric materials can be woven or knitted into textiles or integrated with other energy harvesting devices for improved performance. BaTiO_3_ nanowire-PVC composite fibers can be fabricated by the spinning method, which can be woven onto a PET substrate with cotton threads as the insulating spacers by hand knitting into fabric nanogenerator shown in Figure 17c.^255^ The device can be attached on the human arm to generate output voltage and current of 1.9 V and 24 nA to light up an LCD screen. Different weaving structure can be fabricated by using the unique properties of fibers. A plain weave canvas (2D) and a diagonal interlock (3D) of PVDF fiber by melt spinning was investigated, where the 3D interlock structure nanogenerator exhibited output voltage of 2.3 V, 16 times the output voltage given of 0.14 V by a 2D woven structure.^259^ In addition, a PVDF sheet woven with an elastic hollow tube was also reported as a tactile sensor, which showed a maximum output voltage and power of 51 V and 850 μW, respectively.^260^ As shown in the scheme and cross section of Figure 17d, PVDF film can be integrated with Ni/Cu fabric as flexible piezoelectric nanogenerator by hot-pressing with *d*_33_ coefficient of −32.0 pCN^–1^ exhibiting peak power of 16.83 nWcm^–2^ at an applied impact pressure of 55.5 kPa (6 N), respectively.^256^

To improve the elasticity and breathable properties, fiber or yarn-like piezoelectric materials can be further fabricated into 3D knitted textile devices with two independent layers. A porous spacer can enhance the breathability, compressibility, and recovery of the fabric with excellent mechanical properties. Piezoelectric PVDF with β-phase (∼80%) monofilaments synthesized by melt-spinning extrusion was interconnected with the Ag-coated polyamide multifilament yarn layers served as the top and bottom electrodes to form a 3D spacer yarn.^261^ The output performance of the unique textile structure with 3D spacer produced power density between 1.10 and 5.10 mW/cm^2^ at applied impact pressures between 0.02–0.10 MPa. Instead of using piezoelectric material fibers as a spacer, polyester fiber can also be knitted as a spacer. As shown in Figure 17e, a poled PVDF film was sandwiched inside two multifilament films fabricated as a nanogenerator. It was demonstrated that the space could be regarded as a pressure absorber exhibiting viscoelastic behavior with a maximum output voltage of 25.6 V.^257^ In addition, the effect of density of the monofilaments was investigated, which indicated that higher monofilament densities can give bigger contact areas producing more strain in the piezoelectric PVDF film resulting in higher performance.^257^Figure 17f-g shows PZT hierarchical structure was obtained by using a Ni-yarn as a template, which can be removed by annealing at high temperature of 1000 °C. As shown in Figure 17h,i, after drop-casting PVDF-TrFE on the PZT, PVDF-TrFE/PZT composite material was obtained and used as a pressure sensor with sensitivity of from 72.6 mV kPa^–1^ (0.08–0.85 MPa, *R*^2^ = 0.98) to 186.5 mV kPa^–1^ (0.85–1.3 MPa, *R*^2^ = 0.99), which also worked as human-motion monitoring and provided digital signals via wireless microcontroller unit to a smartphone.^258^

## 7 Application and Outlooks

In light of the preceding discussion, a plethora of piezoelectric energy harvesters have emerged, boasting remarkable attributes such as flexibility, durability, and superior piezoelectric performance. These advancements have paved the way for the tailored design of piezoelectric devices catering to specific application requirements. Extensive efforts have been dedicated to exploring diverse application scenarios, driving innovation in the field. This section aims to comprehensively review the multifaceted applications of piezoelectric technology across various domains, elucidating its profound impact and potential. Furthermore, it provides insights into the ongoing research and development endeavors in the realm of piezoelectric energy harvesters, offering a glimpse into the promising future trajectories of this transformative technology.

**Figure 18:**
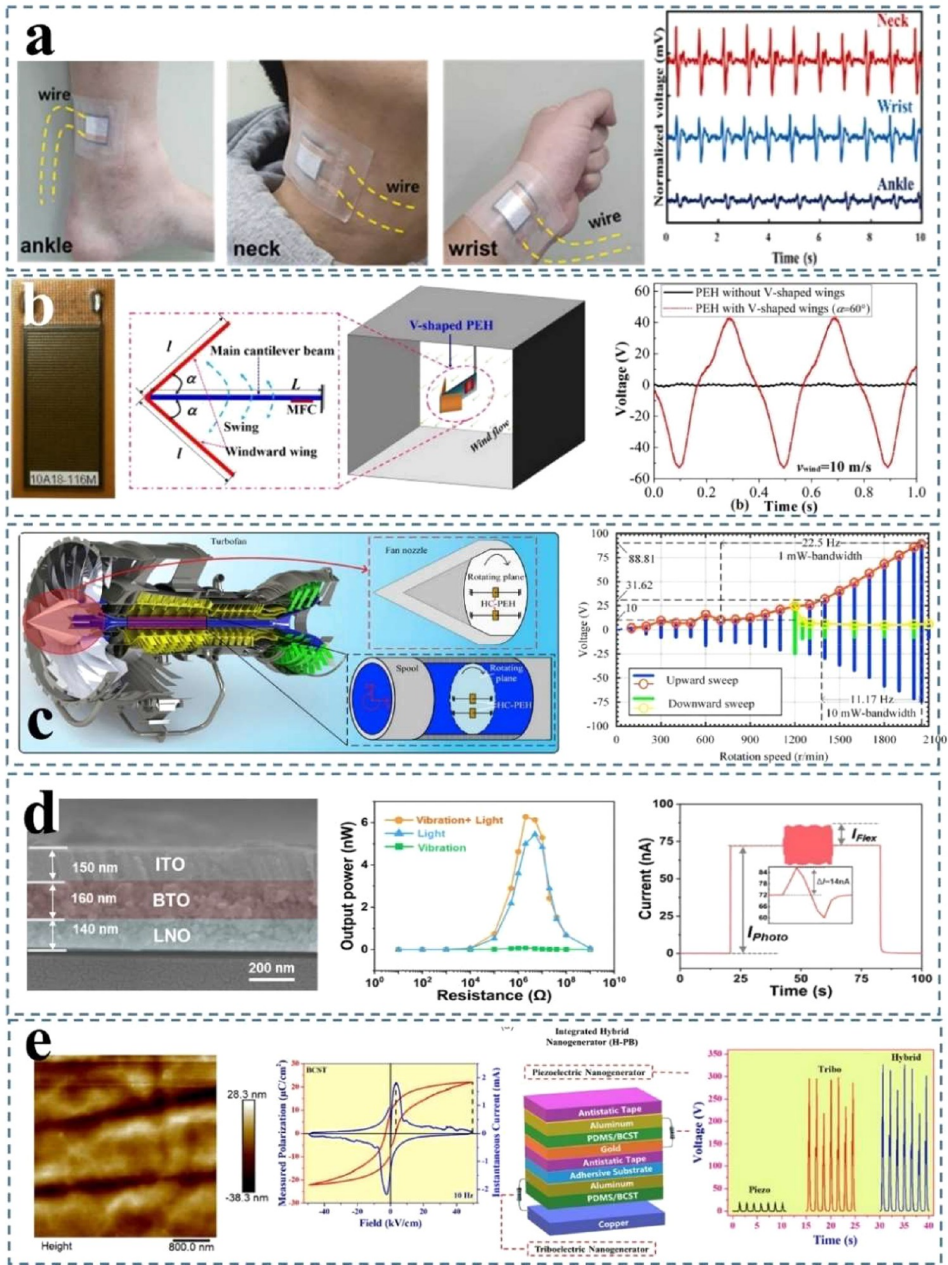
Piezoelectric energy harvester application and hybrid energy harvesters. (a) Output waveforms produced by the piezoelectric device when attached to the neck, wrist, and ankle. Reproduced with permission from ref (262). Copyright 2022 Elservier. (b) Scheme of macrofiber piezoelectric composite (MFC) with V-shaped in wind flow field generating voltage in the angles of 60° at a wind velocity of 10 m/s. Reproduced with permission from ref (265). Copyright 2021 AIP Publishing. (c) Schematic of piezoelectric energy harvester installed to the rotating center of the fan nozzle and spools of the turbofan and the output voltage under at 2050 r/min with the excitation frequency range: 0–35 Hz. Reproduced with permission from ref (267). Copyright 2020 Elservier. (d) Cross-sectional SEM of lanthanum nickelate colloid/BaTiO_3_ hybrid device and the output power and current under vibration and light. Reproduced with permission from ref (278). Copyright 2023 America Chemistry Society. (e) Surface topography of the piezo-tribo hybrid energy harvester based on 0.3Ba_0.7_Ca_0.3_TiO_3_–0.7BaSn_0.12_Ti_0.88_O_3_ (BCST)/PDMS composites and scheme of its piezo-tribo energy harvester generating voltage under an applied force of 2 N. Reproduced with permission from ref (279). Copyright 2023 John Wiley and Sons.

The integration of wearable piezoelectric energy harvesters into clothing has been investigated in the monitoring of human body movements such as finger bending, walking and running, as discussed in section 6. Considering human health and medical application, researchers have explored its biomedical applications in arterial pulse, in vivo implant, deep brain simulation, etc. Figure 18a shows the ZnSnO_3_-carbon nanotube-P(VDF-TrFE) piezoelectric nanogenerator attached on neck, wrist, and ankle with its specific output voltage signal.^262^ The self-powered pulse pressure sensor could be used for instantaneous communication of physiological signals. Cardiac sensors can be implanted inside the body to report heartbeat condition for heart failure. Piezoelectric energy harvesters, PVDF/ZnO/rGO,^263^ were used as self-powered pacemaker on the in vivo experiments on a large animal model with normal physiological activities. A self-powered pacemaker can exempt the patients from surgeries for battery replacement, thereby improving the welfare of the patients. It was reported that Pb(In_0.5_Nb_0.5_)O_3_–Pb(Mg_0.33_Nb_0.67_)O_3_–PbTiO_3_ (PIN–PMN–PT) based deep brain stimulation contracted the forelimb muscle of a mouse resulting in 1.5–2.3 mm displacement of the right paw by bending/unbending device.^264^

From industry/transportation aspects, flexible piezoelectric devices have been utilized to harvest wind energy and torsional vibration induced by internal combustion engines. As shown in Figure 18b, a macrofiber composite (MFC) was used as a piezoelectric energy harvester with a pair of V-shaped windward wings at the angles of 60° on a piezoelectric cantilever beam tested in a wind tunnel, of which the transient alternating voltage ranged from −52 to +43 V at a wind velocity of 10 m/s.^265^ Different piezoelectric wind harvesting system structures can influence the energy conversion such as bluff body, airfoil, flag, wind concentrator and wind turbine structure.^266^Figure 18c is piezoelectric energy harvester (PZT-5H plate) set on the rotational center of the fan nozzle with a maximum output power of 78.87 mW with an external resistance of 100 kΩ at 2050 r/min.^267^ In addition, piezoelectric energy harvesters also hold the potential applications such as tire pressure, vehicle suspension system and smart health structures monitoring.^268−270^

Many renewable energy sources such as light and heat also offer sustainable alternatives for energy harvesting. By implementing energy harvesting to power wireless technologies, large-scale energy demand can be reduced, in particular for battery charging, and new applications such as the IoT can be enabled by locally harvesting power through energy harvesting. Thus, considerable effort has been devoted to the development of energy harvesting devices, encompassing piezoelectric, triboelectric, thermoelectric, and solar cell technologies, all of which find applications across various scenarios.^271−276^ As summarized in Table 4, piezoelectric and triboelectric devices demonstrate promising potential for converting ambient mechanical movement into electricity, unaffected by weather conditions and heat dissipation limitations,^271,272,277^ while thermoelectric devices offer the opportunity to make use of waste heat sources, and solar cell can potentially provide higher power levels, but which are dependent on ambient or solar light sources. Therefore, exploring the optimum application scenarios could lead to better-designed devices for specific purposes.

**Table 4:** Comparison of the Different Types of Devices for Energy Harvesting

	advantages	limitations	applications
piezoelectric energy harvester	low cost, compact size, lightweight, simple structure	low output power and current, limited frequency range	energy harvesting from mechanical movement; self-power portable electronics for movement monitoring; actuators and sensors for measuring pressure, acceleration and vibration, etc.
triboelectric energy harvester	low cost, easy fabrication, high output voltage	high impedance, low output current, dependence on mechanical motion, electrostatic interference	energy harvesting from mechanical movement; self-powered sensor for monitoring, etc.
thermoelectric energy harvester	waste heat recovery, solid-state operation, scalability	limited operating temperature range, heat dissipation challenges, limited power output,	recycle heat waste for power generation, energy-efficient vehicles; integral wearable devices and power sensors; remote monitoring such as weather buoys, etc.
solar cell	abundant solar source, scalability, low carbon footprints	intermittent energy source, high initial costs, complex structure	grid-tied and off-grid power generation; remote power supplier; power portable electronics; large-scale installations, etc.

Taking into account the advantages offered by various types of energy harvesters, hybrid energy harvesters have attracted lots of attention. As shown in Figure 18d, lanthanum nickelate colloid (LNO) coated BaTiO_3_ on ITO was fabricated as hybrid nanogenerator for simultaneously scavenging light and vibration energies, which exhibited a 121% higher current of 85 nA under simultaneous vibration and light illumination under a light intensity of 57 mW/cm^2^ at 405 nm compared with the current peak generated by the photovoltaic effect alone.^278^ Piezoelectric assisted triboelectric energy harvesting can generate larger output voltage. By combining piezoelectric and triboelectric mechanisms, the synergy between piezoelectric and triboelectric effects enables the harvesting of energy from various mechanical motions, such as vibration, bending, and friction, leading to higher power output compared to individual harvesting methods. Figure 18e shows the piezo-tribo hybrid energy harvester based on 0.3Ba_0.7_Ca_0.3_TiO_3_–0.7BaSn_0.12_Ti_0.88_O_3_ (BCST) exhibiting good ferroelectric nature, of which the piezo-tribo energy harvester generating obvious higher rectified voltage of 326 V under applied force of 2 N compared to the corresponding piezoelectric or triboelectric energy harvester.^279^ Hybrid energy harvesting systems can be more reliable and applied in diverse scenarios including wearable devices, structural health monitoring, and IoT sensors due to their ability to harness energy from multiple sources, ensuring continuous power generation even in fluctuating environmental conditions, contributing to the development of sustainable energy solutions and reducing the reliance on traditional power sources. By integrating multiple energy sources, these systems can optimize power generation across various conditions and enhance efficiency and versatility powering a wide array of applications.

## 8 Conclusion

Energy harvesting of ambient mechanical sources and human daily movement has gained lots of attention due to the increasing development of portable microelectronics. Energy harvesters exhibit great application potential in the field of medicine, monitoring, and entertainment such as smart watches, heart-beat monitoring, and walking-step monitoring. In addition, mechanical energy is an abundant and clean source of energy in our daily life, which can be harvested independent of the time and location compared to solar and in some cases thermal energy. Therefore, a range of related research has been reported to investigate techniques to enhance the performance of energy harvesters. In this review, the development progress of piezoelectric energy harvesters was summarized. Various piezoelectric materials can be utilized to harvest energy from mechanical forces by fabricating into energy harvesters. The common fabrication and measurement methods of energy harvesters were also introduced. To further enhance the output performance, the surface of the piezoelectric active layer can be modified using a variety of techniques. Device structure and hybrid energy harvesters design can also be investigated to improve their output performance and mechanical properties. However, facing the future of commercial applications, there are still some important issues related to flexible energy harvesters specially for wearable piezoelectric energy harvesters:

(1) Appropriate electrodes and effective encapsulation methods to integrate the whole energy harvester structure play a key role in the efficiency and durability improvement of energy harvesters. The electrode should satisfy the ability to form a good contact with the active layer and good stability. For sustainability and mass production, the price of the electrodes needs to be considered as well.
(2) The fabrication process of energy harvesting technology is still in the experimental stage with associated complicated processes and high cost. For practical applications, large-scale production of energy harvesters with lower production cost and environmentally friendly procedures and materials should receive more attention in future studies.
(3) The device size of current energy harvesters being developed in research utilizes a small working area in the scale of cm^2^. Large-area flexible devices should be considered to harvest energy from human daily movements (walking, running, etc.). In addition, more research should be focused on the washable performance and durability of the piezoelectric microelectronics, which should also meet the requirements of comfort and breathability.
(4) Suitable storage system to store the power produced by the energy harvesters also needs to be developed. For wearable applications, the storage system also needs to meet the requirement of comfort and durability of the wearable applications.^280−282^
(5) While highly challenging due to the wide potential range of actuation methods and kinetic energy sources, efforts toward standardization of test conditions and parameter reporting would be highly beneficial for the translation of any energy harvester research into commercial applications. At a minimum, the available energy levels of the sources under consideration should be carefully considered to ascertain if they could ever provide sufficient power levels to power even low-level IoT devices. Then input energies/forces/power should be better measured and described so that the overall conversion efficiency could be quantified and alternative approaches and publications could be compared. Certainly, minimum levels of parameter reporting such as peak power levels, as called for many years previously,^283^ are still lacking in some works and should definitely always be included. Through this the many highly ambitious claims for the potential for energy harvesters can be tested with concrete data.

Flexible electronics have been the subject of much research, and significant advancements have been realized. Particularly wearable piezoelectric technology has developed with an enormous and expanding range of uses. Piezoelectric materials can be combined with portable devices, textiles, and other flexible applications which, if proven effective through rigorous testing and reporting, have the potential to revolutionize how we power the ever increasing range of portable devices in the future.
